# Research Progress of Circular RNA in Gastrointestinal Tumors

**DOI:** 10.3389/fonc.2021.665246

**Published:** 2021-04-15

**Authors:** Na Fang, Guo-Wen Ding, Hao Ding, Juan Li, Chao Liu, Lu Lv, Yi-Jun Shi

**Affiliations:** ^1^ Department of Oncology, The Affiliated People’s Hospital, Jiangsu University, Zhenjiang, China; ^2^ Department of Thoracic and Cardiovascular Surgery, The Affiliated People’s Hospital, Jiangsu University, Zhenjiang, China; ^3^ Department of Respiratory, The Affiliated People’s Hospital, Jiangsu University, Zhenjiang, China

**Keywords:** circular RNA, circRNA, gastrointestinal tumors, pre-mRNA, miRNA

## Abstract

circular RNA (circRNA) is a closed ring structure formed by cyclic covalent bonds connecting the 5’-end and 3’-end of pre-mRNA. circRNA is widely distributed in eukaryotic cells. Recent studies have shown that circRNA is involved in the pathogenesis and development of multiple types of diseases, including tumors. circRNA is specifically expressed in tissues. And the stability of circRNA is higher than that of linear RNA, which can play biological roles through sponge adsorption of miRNA, interaction with RNA binding protein, regulation of gene transcription, the mRNA and protein translation brake, and translation of protein and peptides. These characteristics render circRNAs as biomarkers and therapeutic targets of tumors. Gastrointestinal tumors are common malignancies worldwide, which seriously threaten human health. In this review, we summarize the generation and biological characteristics of circRNA, molecular regulation mechanism and related effects of circRNA in gastrointestinal tumors.

## Introduction

Gastrointestinal tumors such as gastric cancer, esophageal cancer, colorectal cancer, pancreatic cancer, hepatocellular carcinoma and gallbladder cancer are common malignancies worldwide, which seriously threaten human health. The occurrence and progression of carcinomas are related to multiple factors. It is reported that circRNAs are associated with cancers, including gastrointestinal cancers (1–4).

circular RNA (circRNA) is a type of long non-coding RNA. In 1976, Sanger et al. found that the pathogenic plant virus was a single-stranded covalently closed circRNA molecule, but scientists considered that it was connected by host cell enzymes rather than formed by reverse splicing (5). circRNA formed by reverse splicing was first reported in the 1890s (6–9). However, only a few circRNAs were discovered at that time due to technical limitations. Until the 21st century, with the vigorous development of second-generation sequencing (NGS) technology and bioinformatics, it is gradually realized that circRNA is a type of non-coding RNA with prevalent distribution in nature as well as abundant and diverse expression (10). This renders a more intensive understanding of circRNA and in-depth researches on its formation process and mechanism.

## Generation of circRNA

**Figure 1:**
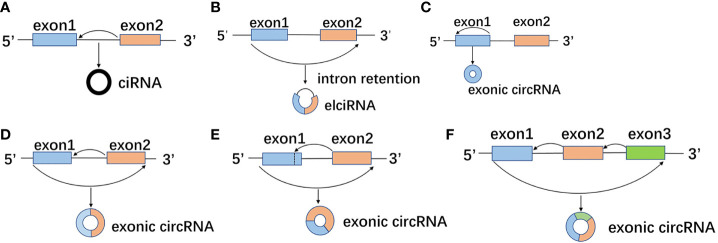
Different splicing sites and compositions form different circRNAs. **(A)** The splicing sites of pre-mRNA during the formation of intron circRNA (ciRNA) by intron circularization. **(B)** The splicing sites of pre-mRNA during exon and intron cyclization to form exon-intro circRNA (EIciRNA). **(C-F)** The splicing sites of pre-mRNA in the process exon cyclization to form exonic circRNA (ecircRNA).

circRNA is formed by reverse splicing of pre-mRNA, and the same pre-mRNA can generate multiple circRNAs with different compositions due to the difference of splicing sites (11). The generated circRNA can be classified into three types according to the different composition: intronic circRNA (ciRNA) formed by intron cyclization (**Figure 1A**) (12), and exon-intro circRNA (EIciRNA) formed by exon and intron cyclization (**Figure 1B**) (13), and exonic circRNA (ecircRNA) formed by exon cyclization (**Figures 1C–F**) (14), among which exon circRNA is the most common. Most human endogenous circRNA contains multiple exons, with two or three exons as the most common. Each exon generally contains 112 to 130 nucleotides. There is also single exon formed by reverse splicing, which generally requires a median exon length of 353 nucleotides (15).

**Figure 2:**
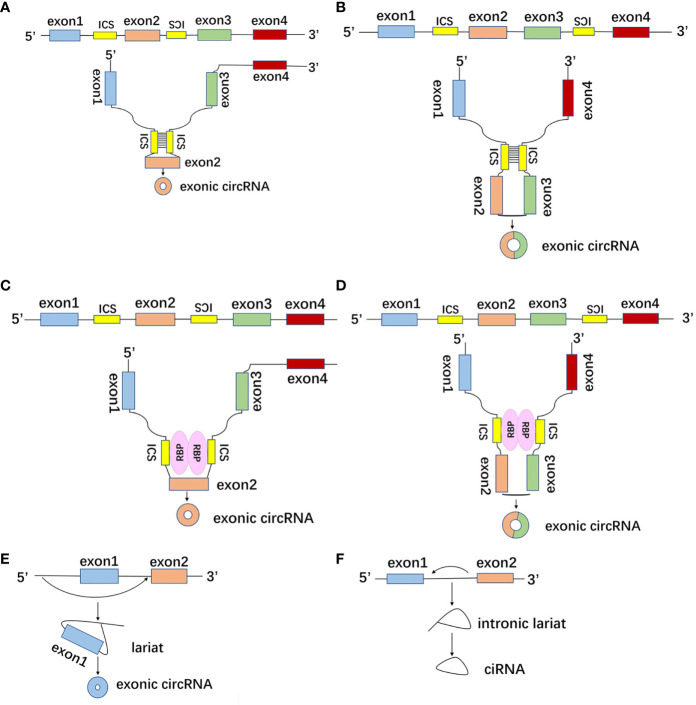
The formation modes of circRNA. **(A, B)** CircRNAs with intronic complementary sequence (ICS) of pre-mRNA can make the distal intron splicing sites closer in space to promote reverse splicing and ICS pair-driven cyclization. **(C, D)** CircRNAs with double-stranded RNA binding domain (dsRBD) can bind and pair with ICS to make the splice site closer, as well as facilitate reverse splicing to increase circRNAs formation. **(E)** In the exon skipping event, the exon lariat formed by the covalently combined splice acceptor and splice donor provided by the exon. **(F)** Intronic lariat-driven cyclization was caused by intron removal during the pre-mRNA splicing process.

Regardless of the splicing method, circRNA is formed by reverse splicing *via* spliceosome on the downstream 5’-splicing site and upstream 3’-splicing site of pre-mRNA and subsequent formation of 3’,5’-phosphodiester bond as well as cyclization (16). The formation modes of circRNA include intronic complementary sequence (ICS) pair-driven cyclization, RNA binding protein (RBP)-driven cyclization and lariat-driven cyclization (17). First of all, ICS of pre-mRNA can make the distal intron splicing sites closer in space to promote reverse splicing (**Figures 2A, B**) (18). Secondly, RBP can regulate the formation of circRNA by combining with ICS (**Figures 2C, D**). RBP usually contains a double-stranded RNA binding domain (dsRBD), and dsRBD can bind and pair with ICS. Nuclear factor 90 (NF90) and nuclear factor 110 (NF110) encoded by the ILF3 gene both contain dsRBD, which can promote the formation of circRNA by directly binding to the intronic reverse repeat Alu element (19, 20). ICS of SEPT9 can bind to EIF4A3 to increase the production of circSEPT9 (21). Quaking binds to ICS to make the splice site closer to facilitate reverse splicing to increase circRNAs formation (22). Thirdly, in the exon skipping event, the exon lariat formed by the covalently combined splice acceptor and splice donor provided by the exon is another formation of circRNA (**Figure 2E**) (23). Additionally, the formation of intron lariat caused by intron removal during the pre-mRNA splicing process can give rise to circRNA (**Figure 2F**) (12).

Then, what are the factors affecting the formation of circRNA? Firstly, polymerase II plays an important role in reverse splicing. Analysis of the transcription elongation rate of human cellular polymerase II showed that the average transcription elongation rate of genes that can produce circRNA is higher than that of genes that cannot produce circRNA. The high transcription elongation rate renders transcription of more downstream genes and increases ICS matching that skipps exon, therefore, reverse splicing is more likely to form circRNA (10). After depletion cleavage/polyadenylation terminates the inhibitory effect of RNA polymerase II, the level of circRNA also increases (24). Moreover, reverse splicing and canonical splicing are in a competitive relationship. When the canonical splicing speed becomes slow or the splicing complex is consumed, the level of circRNA would increase, which is associated with the transformation from canonical splicing to reverse splicing (25).

## Detection Method and Research Technology of circRNA

RNA sequencing and gene chip technology have relatively good sensitivity analysis and quantitative accuracy of circRNA detection, however, with expensive cost. Northern blot and reverse transcription polymerase chain reaction are also simple and effective methods to detect circRNA, however, with relatively low sensitivity analysis and quantitative accuracy. Quantitative reverse transcription polymerase chain reaction can be performed if rapid and accurate detection of the relative expression abundance of circRNA is required, whose sensitivity analysis and quantitative accuracy are better, and the cost is relatively low. The sensitivity analysis and quantitative accuracy of droplet digital PCR and NanoString Technologies nCounter assays to detect circRNA are extremely good, however, they are rarely used due to special equipment and relatively expensive cost (26).

Two-dimensional denaturing polyacrylamide gel electrophoresis can be used to assess whether RNA is circular (27). The cellular localization of circRNA requires FISH technology (13) and nuclear and cytoplasmic separation (14). Bioinformatics analysis and RNA-seq can be used to predict and to analyze the interaction between circRNA and miRNA. Bioinformatics analysis and identification of RNA pulldown and mass spectrometry can be used to predict and to analyze the interaction between circRNA and other proteins. RNA immunoprecipitation related assays and dual luciferase reporter gene assay can be used to validate their interaction.

## Biological Characteristics of circRNA

In recent years, the continuous in-depth studies of circRNA have been revealed diverse noteworthy characteristics of circRNA.

### Stability of circRNA

circRNA has a closed loop structure with 3’,5’-phosphodiester bonds, without 5’˜3’ polarity or poly A tail, which makes them more stable than linear RNA and not easily degraded by RNase R (28). In addition, during viral infection, circRNA is almost completely degraded by RNase L (29). The ribonuclease complex RNase P/MRP can degrade m6A-modified circRNA through the m6A reader protein YTHDF2 and HRSP12 (30). Another study has found that the combination of AGO2 protein and miRNA can mediate the degradation of circRNA. CDR1as contains miR-671 binding sites, and their binding can mediate the degradation of CDR1as by AGO2 (31). Meanwhile, miR-7 can also promote the degradation of circRNA by recruiting miR-671 (32). AGO2 can also mediate the degradation of circFilip11 by miRNA-1224 (33). Moreover, circRNA can also enter exosomes or extracellular vesicles, which can be eliminated by the export of active substances (34). Overall, circRNA is more stable than linear RNA, with an average half-life of 18.8-23.7 h, while the average half-life of homologous linear RNA is only 4.0-7.4 h (35).

### Localization of Characteristics of circRNA

ciRNA and EIciRNA are mainly distributed in the nucleus of eukaryotes, and ecircRNA is mainly distributed in the cytoplasm (12–14). Partially-length circRNA could be transported from the nucleus to the cytoplasm, and different species have different requirements for the length of circRNA from the nucleus to the cytoplasm (36). In drosophila and human cells, Hel25E protein family is a key regulator that mediates the transport of circRNA from the nucleus to the cytoplasm. The ATP-dependent RNA helicase Hel25E (also known as WM6) in drosophila melanogaster mediates the export of long-chain circRNA with over 800 nucleotides in length. The homologous protein UAP56 (DDX39B) of Hel25E in human cell mainly mediates the nuclear export of long-chain circRNA with over 1300 nucleotides in length, while URH49 (DDX39A) mainly mediates the nuclear export of short-chain circRNA with less than 500 nucleotides in length. The amino acid sequence differences of the Hel25E protein family lead to its recognition of circRNA molecules of different sizes (37). YTHDC1 can regulate the nuclear export of m6A-modified mRNA (38). Chen et al. found that m6A-modified circNSUN2 can bind to YTHDC1 to promote the nuclear export of circNSUN2 (39).

### Type and Expression Abundance of circRNA

High-throughput sequencing analysis has revealed the expression of multiple types of circRNA in fungi, protists, plants, worms, fish, insects and mammals (40–45). By identifying the transcriptomes of normal tissues and tumor tissues of humans and other animals, Zhao et al. identified most of the full-length sequences of circRNAs, and compiled the circRNA database (circAtlas) (46). The circAtlas database presently contains circRNAs from homo sapiens, macaca mulatta, mus musculus, rattus norvegicus, sus scrofa and gallus gallus. There were 421,501 types of circRNAs from 259 human samples, 169618 types of circRNAs from 80 macaque samples, and 175,273 types of circRNAs from 113 mouse samples.

circRNA is abundantly expressed in mammalian brain tissue (47–49), which is also enriched in human red blood cells and platelets (50). Notably, although the efficiency of reverse splicing is not high, the accumulation of circRNA is considerable due to its stability, therefore, the expression level of circRNA can be higher than its homologous linear mRNA. The stable abundance of circRNA is a balanced consequence of circRNA formation, nuclear output and turnover efficiency (10).

### circRNA Is Highly Conservative and Expresses Specifically at the Stage of Tissue Development

Despite various types of circRNA, most types of circRNA are extremely conservative in evolution and among different species (51). The conservation of circRNAs among different species indicates that circRNAs are not by-products of precursor mRNA splicing, suggesting circRNA is extremely important in certain biological processes. circRNAs are specifically expressed at the stage of tissue development, and are involved in innate immunity, development of the nervous system, metabolism of hormones in the body, as well as tumorigenesis and tumor progression (52–55).

## The Influences of Life Style, Nutrition, Diet, Environment and the Microbiome on circRNAs.

Based on the heterogeneity of disease, molecular pathological characteristics and epidemiological study design method, molecular pathological epidemiology analyzes the impact of molecular level changes caused intrinsic factors and extrinsic factors (such as life style, nutrition, diet, environment and the microbiome) on the occurrence, development, prognosis and outcome of the disease (56, 57). Studies have found that circRNA can be affected by different extrinsic factors. Chemical contamination in the environment is known to cause abnormal circRNA expression through multiple exposure routes (58). Fatty liver may result from excessive triglyceride uptake and production by the liver or by a secretory defect (59). The aberrant expression of circScd1 affects the extent of hepatocellular lipidosis in nonalcoholic fatty liver disease and promotes fatty liver disease *via* the JAK2/STAT5 pathway (60). Plasmatic circRNA MBOAT2 demonstrated a significantly lowered level 24 h after the marathon (61). CircNF1-419 improves the gut microbiome structure and function in AD-like mice (62). Cancers are complex diseases which are related to the above exogenous factors (63–65). There are also some relationships between circRNAs and exogenous factors in cancers. Gut microbiota regulate tumor metastasis *via* circRNA/miRNA networks (66). Zhang et al. have found that the expression of circ-DB in plasma exosomes of in hepatocellular carcinoma (HCC) patients with high body fat rate is up-regulated. After being taken up by HCC cells, exosomal circ-DB inhibits the expression level of miR-34a and activates deubiquitination-related USP/Cyclin A2 signaling pathway, thereby promoting the proliferation of HCC and attenuating cell DNA damage (67).

## The Mechanism of circRNA in Gastrointestinal (GI) Tumors

In recent years, circRNA has been widely investigated in multiple diseases (68–70), including GI tumors (**Tables S1**–**S7**). Despite act as miRNA sponge, circRNAs can function as interaction with RNA binding protein, regulation of gene transcription, the mRNA and protein translation brake, and translation of protein and peptides in GI tumors.

### As miRNA Sponge

**Figure 3:**
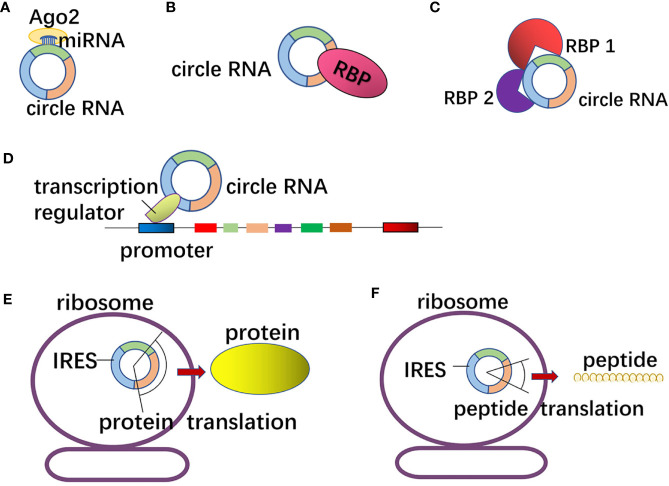
The mechanism of circRNA in gastrointestinal tumors. **(A)** CircRNAs can act as miRNA sponge or decoys as well as regulate the function of downstream mRNA. **(B)** CircRNAs with RNA binding protein (RBP) binding motifs may sponge or decoy the RBPs and regulate their functions. **(C)** A few circRNAs can combine with several RBPs and function as protein scaffolds to affect the tumor progressions. **(D)** Some circRNAs are involved in gene transcription regulation by recruiting the transcription regulators to influence promoters. **(E, F)** CircRNAs containing internal ribosome entry site (IRES) elements and AUG sites may act as templates as well as be translated.

circRNA can act as miRNA sponge in GI tumors (**Figure 3A**). circLPAR3 is highly expressed in esophageal squamous cell carcinoma (ESCC) tissues and cells, which can upregulate the expression of c-MET through sponge adsorption of miR-198 to increase the migration and invasion of ESCC (55). circCCDC9, with low expression in gastric cancer tissues and cells, can attenuate the inhibitory effect on the target gene CAV1 after adsorbing miR-6792-3p, thereby inhibiting the proliferation, migration and invasion of gastric cancer cells (71). circCAMSAP1 is highly expressed in colorectal cancer tissues than in normal tissues, and its expression is significantly lower in the plasma of colorectal cancer patients than that before surgery. circCAMSAP1 sponges miR-328-5p to weaken its inhibitory effect on transcription factor E2F1, thereby promoting proliferation of colorectal cancer cells (72). circBFAR, with high expression in pancreatic ductal adenocarcinoma, up-regulates the expression of mesenchymal-epithelial transformation (EMT) through sponge adsorption of miR-34b-5p to phosphorylate Akt at Ser 473, which further activates MET/PI3K/Akt signal transduction pathway to promote the proliferation, migration and invasion of pancreatic ductal adenocarcinoma (73). Circular RNA cSMARCA5 with low expression in HCC tissues and cells, can promote the expression of tumor suppressor gene TIMP3 by sponge adsorption of miR-17-3p and miR-181b-5p to suppress the proliferation and migration of HCC (74).

All of the above findings suggest that circRNA can act as a sponge to adsorb one or more miRNAs to subsequently affect the expression of downstream target genes, thereby affecting the biological functions of tumor cells, with potential therapeutic value.

### Interaction With RNA Binding Protein (RBP)

circRNA can affect the function of downstream target genes through competitive binding with RBP in GI tumors (**Figure 3B**) (75). circGSK3β is highly expressed in ESCC, and circGSK3β can bind to GSK3β protein to prevent β-catenin from phosphorylation and degradation, to affect the Wnt/β-catenin pathway, thereby promoting the migration and invasion of ESCC (76). circPTK2 is highly expressed in cancer tissues and serum of patients with colorectal cancer (CRC). To be specific, circPTK2 can affect the phosphorylation and expression level of Vimentin by binding to Ser38, Ser55 and Ser82 of Vimentin, which can promote EMT, inhibit apoptosis and enhance cell proliferation, migration and invasion of CRC cells (77). circAGO2 is significantly elevated in gastric cancer tissues. circAGO2 interacts with human antigen R (HuR) protein to promote the activation and enrichment of 3’-UTR of target genes, to prevent target genes from binding to AGO2, and to decrease the formation of AGO2-miRNA complex, thereby promoting the proliferation, invasion and metastasis of gastric cancer cells (78). circ-FOXP1 is highly expressed in gallbladder cancer (GBC) tissues and cells. By interacting with PTBP1 protein, circ-FOXP1 can protect PKLR mRNA from decay, promote cell proliferation, migration, invasion and inhibit apoptosis of GBC (79).

circRNA can combine with several RBPs to act as a protein scaffold (**Figure 3C**). In metastatic CRC, m6A-modified circNSUN2 combines with IGF2BP2 protein and HMGA2 protein to form a ternary complex to promote liver metastasis of CRC (39). circMRPS35 is lowly expressed in gastric cancer, and it can serve as a protein scaffold to recruit histone acetyltransferase KAT7 to the promoters of FOXO1 and FOXO3a genes to further cause H4K5 acetylation, which further activate the transcription of FOXO1 and FOXO3a and trigger the expression of their downstream targets (p21, p27, Twist1 and E-cadherin genes), thereby inhibiting the proliferation and invasion of gastric cancer cells (80).

### Regulation of Gene Transcription

Li et al. have found that in cervical cancer Hela cells, circEIF3J, circPAIP2 interact with Pol II and U1 snRNP to bind to the parent gene promoter to promote parental gene transcription (13). However, the above regulatory pathway of gene transcription by circRNA has not been reported in GI tumors, while circRNA can regulate gene transcription through other pathways (**Figure 3D**).

circRHOT1 is highly expressed in HCC tissues and cells. circRHOT1 recruits TIP60 to the NR2F6 promoter and initiates NR2F6 transcription, promotes the expression of NR2F6, and enhances the proliferation, migration and invasion of HCC cells *via* the NOTCH2 signaling pathway (81). circERBB2 is highly expressed in GBC tissues and is mainly located in the nucleoli of GBC cells. It can bind to PA2G4 to regulate the nuclear localization of PA2G4. The binding of circERBB2 to PA2G4 can regulate the expression of TIFIA to regulate rDNA transcription, thereby promoting the proliferation of GBC cells (82). circ-DONSON is highly expressed in gastric cancer tissues and cells. It can interact with the important subunit SNF2L of the NURF complex, recruit NURF to the SOX4 promoter to initiate its transcription, promote the proliferation, migration and invasion and inhibit apoptosis of gastric cancer cells (83). circITGA7, with low expression in CRC, can increase the expression of NF1, a negative regulator of the Ras1 pathway after sponge adsorption of miR-370-3p, which can inhibit the Ras pathway, decrease the expression of RREB1, promote linear ITGA7 transcription to increase the expression of linear ITGA7, while overexpression of circITGA7 can inhibit the proliferation, migration and invasion of CRC cells (84).

### The mRNA and Protein Translation Brake

In GI tumors, a few circRNAs can interfere with the translation of mRNA or protein. circBACH1 is significantly elevated in liver cancer tissues and cells. It can bind to HuR and promote the translocation of HuR from the nucleus to the cytoplasm. In HCC, circBACH1 can enhance the inhibitory effect of HuR on p27 translation and accelerate the cell cycle from G0/G1 phase to S phase, thereby promoting the proliferation of HCC (85). circ-MALAT1 is highly expressed in cancer stem cells (CSCs) of HCC tissues. circ-MALAT1 binds to ribosomes through the internal ribosome entry site (IRES) and binds with PAX5 mRNA through 11 bases to form circ-MALAT1, ribosome and PAX5 mRNA ternary complex, which prevents the translation of PAX5 and promotes the self-renewal of CSCs (86).

### Protein or Peptide Translation

Studies have found that endogenous circRNA containing IRES and ribosome binding sites (AUG) has protein coding capacity (87). The translation mechanism of circRNA is different from that of ordinary linear mRNA, and it is initiated by ribosome scanning. Several circRNAs can act as templates for translation in GI tumors (**Figures 3E, F**). The translation mechanism of circRNA is similar to the cap-independent translation pathway. Studies have shown that m6A modification can drive circRNA translation in a cap-independent manner (88).

circPPP1R12A is highly expressed in gastric cancer tissues and cells. The short 216 nt small open reading frame of circPPP1R12A has the potential to peptide encoding (containing 73 amino acids). The encoded circPPP1R12A-73aa peptide can activate the hippo-YAP signaling pathway and enhance the proliferation, migration and invasion of gastric cancer cells (89). circFNDC3B is lowly expression in colon cancer tissues, and it can encode a new protein circFNDC3B-218aa. circFNDC3B-218aa can decrease the expression of Snail to promote the antitumor effect of FBP1 in colon cancer, to suppress the proliferation, invasion, migration and EMT of colon cancer cells (90). circβ-catenin is highly expressed in HCC tissues, which can encode a 370-amino acid protein (known as β-catenin-370aa). β-catenin-370aa competitively binds to GSK3β to prevent the binding and phosphorylation of GSK3β and β-catenin, thereby antagonizing the GSK3β-induced degradation of β-catenin and activating the Wnt pathway to promote the growth and metastasis of HCC cells (91).

Collectively, circRNA regulates GI tumors through various mechanisms, including miRNA sponge adsorption, interaction with RBP, regulation of gene transcription, the mRNA and protein translation brake, and translation of protein and peptides. These mechanisms are not completely separated, but interactive.

## Biological Functions of circRNA in GI Tumors

### Regulation of Proliferation, Migration, Invasion and Apoptosis of Tumor as Well as Self-Renewal of CSCs

There are extensive studies concerning the roles of circRNA the regulation of tumor proliferation, migration, invasion and apoptosis. In addition, circRNA can also affect the self-renewal of CSC. circHuR expression is down-regulated in gastric cancer tissues and lines. circHuR interacts with CNBP protein (CCHC-type zinc finger nucleic acid-binding protein) and inhibits the binding of CNBP to the HuR promoter, thereby leading to HuR down-regulation to further inhibit the proliferation, invasion and metastasis of gastric cancer cells (92). circLgr4 is highly expressed in CRC tissues and CSCs, with peptide-coding functions. The peptide encoded by circLgr4 interacts with Lgr4 and is activated by Lgr4, to further promote the activation of Wnt/β-catenin signal, which can promote the invasion of CRC cells and self-renewal of CSCs (93). circZMYM2 is overexpressed in pancreatic cancer tissues and cells. It increases the expression of JMJD2C by binding to miR-335-5p, promotes the proliferation and invasion as well as inhibits apoptosis of pancreatic cancer cells (94). hsa_circ_0016788 is up-regulated in HCC tissues and cell lines. It can increase the expression of CDK4 by binding to miR-486, promote the proliferation and invasion as well as inhibit apoptosis of HCC cells (95). The expression of circ-CCAC1 derived from ERBB2 is increased in cholangiocarcinoma cells. circ-CCAC1 up-regulates the expression of transcription factor YY1 by competitively binding to miR-514a-5p in tumor cells. YY1 promotes its transcription by directly binding to CAMLG promoter, thereby promoting the proliferation, migration and invasion of GBC cells (96).

### Regulation of the Radiotherapy and Chemotherapy Response of Tumor Cells

In addition to affecting tumor proliferation and metastasis, circRNA can also affect the sensitivity of tumor cells to radiotherapy and chemotherapy. Then, does circRNA still have this function in GI tumors?

circVRK1 is lowly expressed in ESCC tissues and cells. It can positively regulate PTEN, inhibit the activity of PI3K/AKT signaling pathway, suppress proliferation, migration and EMT of ESCC cells as well as reverse radiation therapy resistance by adsorbing miR-624-3p (97). Has_circ_001680 is highly expressed in CRC tissues. It can induce irinotecan resistance by regulating the miR-340 to affect the target gene BMI1 (98).

The activity of key metabolites of autophagy is associated with the drug resistance of tumors, and circRNA can regulate the drug sensitivity of tumor cells by affecting autophagy (99). circRACGAP1 can sponge miR-3657 and further up-regulate the expression level of ATG7 by competitively inhibiting miR-3657 activity. Both endogenous and exogenous knockdown of circRACGAP1 expression can increase the sensitivity of gastric cancer cells to apatinib by suppressing autophagy, and knockdown of circRACGAP1 can decrease the toxicity of apatinib and enhance its therapeutic effect on gastric cancer (100). circCUL2 is lowly expressed in gastric cancer tissues and cells. circCUL2 can increase the expression of ROCK2 and p62, inhibit the expression of autophagic marker LC3 and Beclin1 by adsorbing miR-142-3p, which can inhibit autophagy and improve cisplatin sensitivity of cisplatin-resistant GC cells (101).

ABC transporter can transport chemotherapeutics to the extracellular compartment, specific organelles and exosomes. Therefore, the drug retention in the cellular vesicles and compartments can increase drug efflux, which reduces the drug concentration to cause chemotherapeutic resistance of tumor (100). circFBXO11 is highly expressed in HCC tissues. It can sponge miR-605 to decrease its inhibition of FOXO3 protein, and increased FOXO3 expression targets the promoter region of ABCB1 to accelerate its expression, thereby increasing the anti-oxaliplatin ability of HCC (102).

### Effects on Immune Therapy

Immune function is crucial to tumorigenesis and tumor progression. circRNA can affect immune function. circ_0000977 in pancreatic cancer can adsorb miR-153 to affect the expression of HIF1A and ADAM10 as well as regulate the immune escape of pancreatic cancer cells mediated by HIF1A (103). circMET is highly expressed in HCC. It can affect the expression of Snail/dipeptidyl peptidase 4 (DPP4)/CXCL10, induce EMT, enhance the immunosuppression of the tumor microenvironment and promote HCC progression after adsorbing miR-30-5p (104). The HCC cell-secreted exosomes circUHRF1 can inhibit the function of NK cells, promote the immune escape of HCC cancer, and increase the resistance of anti-PD1 immunotherapy through the miR-449c-5p/TIM-3 pathway, which provide a novel therapeutic approach for HCC patients, that is, targeting circUHRF1 (105).

### circRNAs and Tumor Microenvironment in GI Tumors

The tumor microenvironment (TME) consists of extracellular matrix components, endothelial cells, stromal cells, immune cells, vasculature and various signaling entities. TME, nourished by the vasculature, is an indispensable condition for metastatic tumor cell growth. It is important that endothelial cells of the TME which associate with angiogenesis and tumors metastasis. VEGF is the most important regulatory factor in angiogenesis (106). It is a mitogenic factor to promote the proliferation of endothelial cells and angiogenesis. circ_0072088 is overexpressed in ESCC cells and tissues. It can sponge miR-377 to attenuate the inhibitory effect of miR-377 on VEGF expression, which could elevate the expression of VEGF, thereby promoting migration and invasion of ESCC (107). Furthermore, exosomal circ-IARS is overexpressed in pancreatic cancer cells. It can promote the permeability of the vessel wall to accelerate tumor metastasis. The circ-IARS can sponge miR-122 and enhance the activity of Ras homolog gene family, member A (RhoA), which restrains tight junction ligand-protein Zonula occludens-1(ZO-1) and promotes endothelial monolayer permeability, thereby promoting tumor development (108). In conclusion, circRNA may plays a crucial role in the TME.

## Exosomal circRNAS in GI Tumors

Exosomes are extracellular vesicles (EV) secreted by most eukaryotic cells and involved in intercellular communication. The components of exosomes include protein, DNA, RNA, etc. After circRNA is exported from exosomes and released in recipient cells, it plays a critical role in regulating tumor growth, metastasis and angiogenesis during tumor development (109). Then, what is the expression and role of exosomal circRNA in GI tumors? The expression of circPAGRAL is significantly up-regulated in CRC cells treated with tumor-derived exosomes, which promotes the proliferation, migration and invasion of CRC cells by modulating the miR-142-3p/miR-506-3p-TGF-β1 axis (110). The high expression of circSHKBP1 in gastric cancer, can be delivered by exosomes and promote the proliferation, migration and invasion of gastric cancer cells *in vitro via* sponge adsorption of miR-582-3p and binding to HSP90 (111). Exosome-delivered circ_MMP2 in HCC promotes HCC metastasis by up-regulating MMP2 (112). After endothelial cells receive extracellular vesicles (mainly exosomes) carrying circ-CCAC1 released by cholangiocarcinoma cells, circ-CCAC1 can bind to EZH2 in endothelial cells and prevent the nuclear translocation of EZH2, weakening EZH2-mediated H3K27me3 modification of SG3GL2 promoter region. The SG3GL2 promoter is activated and leads to up-regulated expression of SG3GL2 to inhibit the expression of ZO-1/Occludin, to weaken the tight junction of endothelial cells, to increase permeability and to promote infiltration and migration of tumor cells (96).

## Clinical Application

### The Diagnostic and Prognostic Roles of circRNAs in GI Tumors

At present, a variety of circRNAs have been found to have abnormal expression in plasma, serum and exosomes in ESCC, gastric cancer, HCC and CRC (113), which makes circRNA as a promising marker for liquid biopsy of GI tumors. For instance, the abnormal expression of circGSK3β (76), hsa_circ_0001946 and hsa_circ_0043603 (114) in the plasma of ESCC patients is associated with the prognosis of ESCC patients. circSHKBP1 is highly expressed in the serum of patients with gastric cancer, which is also correlated with the prognosis of patients with gastric cancer (111). The abnormal expression of hsa_circ_0051443 (115), hsa_circ_100338 (116), circ-ZEB1.33 (117) in the serum of HCC patients is related to the prognosis of HCC patients. The abnormal expression of hsa_circ_0000370 in the blood of CRC patients is associated with the prognosis of CRC patients (118). The expression of circ-LDLRAD3 in the plasma of patients with pancreatic cancer is high, which is also related to lymph node metastasis, vascular invasion and clinical stage (119). The high stability and specific expression of blood circRNAs render it as an ideal biomarker for liquid biopsy.

### circRNA as a Possible Therapeutic Target for GI Tumors

Jost et al. have found that circRNA, which targets miR-122, isolates miR-122 from HCV through sponge action, thereby effectively suppressing and decreasing the replication and spread of HCV (120). In GI tumor studies, many animal experiments have revealed that interference with circRNA expression can inhibit the occurrence and progression of tumors. For instance, circLPAR3 is highly expressed in ESCC. Nude mice injected with ESCC cells with low expression of circLPAR3 had significantly lower lung metastasis rate than those injected with control cells (tail vein model) (55). In addition, MET inhibitors can inhibit circBFAR-mediated PDCA in nude mice (73).

## Conclusions and Perspectives

circRNA, a novel type of non-coding RNA, has been a research hotspot in GI tumors in recent years. Despite gratifying results, there are still many problems to be solved, such as the generation mode, influencing factors, degradation, biological effects and mechanisms of circRNA in GI tumors. A large number of in-depth studies are required for circRNA as a biomarker and therapeutic target for GI tumors as well as its clinical application.

## Author contributions

Y-JS provided direction and guidance throughout the preparation of this manuscript. NF wrote and edited the manuscript. G-WD, HD and JL reviewed and made significant revisions to the manuscript. CL and LL collected and prepared the related papers. All authors contributed to the article and approved the submitted version.

## Funding

This work was supported by the Research Fund of The Affiliated People’s Hospital of Jiangsu University (grant nos. Y2019015).

## Conflict of Interest

The authors declare that the research was conducted in the absence of any commercial or financial relationships that could be construed as a potential conflict of interest.

## Abbreviations

circRNAs, Circular RNAs; RBP, RNA binding protein; ICS, Intronic complementary sequence; dsRBD, Double-stranded RNA binding domain; GI, Gastrointestinal; ESCC, Esophageal squamous cell carcinoma; EMT, Mesenchymal-epithelial transformation; HCC, Hepatocellular carcinoma; CRC, Colorectal cancer; GBC, Gallbladder cancer; IRES, Internal ribosome entry site; CSCs, Cancer stem cells.

## References

[1] Naeli P Pourhanifeh MH Karimzadeh MR Shabaninejad Z Movahedpour A Tarrahimofrad H. Circular RNAs and gastrointestinal cancers: Epigenetic regulators with a prognostic and therapeutic role. Crit Rev Oncol Hematol (2020) 145:102854. 10.1016/j.critrevonc.2019.102854 31877535 PMC6982584

[2] Ashrafizadeh M Zarrabi A Hashemipour M Vosough M Najafi M Shahinozzaman M. Sensing the scent of death: Modulation of microRNAs by curcumin in gastrointestinal cancers. Pharmacol Res (2020) 160:105199. 10.1016/j.phrs.2020.105199 32942019

[3] Shafabakhsh R Arianfar F Vosough M Mirzaei HR Mahjoubin-Tehran M Khanbabaei H. Autophagy and gastrointestinal cancers: the behind the scenes role of long non-coding RNAs in initiation, progression, and treatment resistance. Cancer Gene Ther (2021) 1:1–27. 10.1038/s41417-020-00272-7 33432087

[4] Pourhanifeh MH Vosough M Mahjoubin-Tehran M Hashemipour M Nejati M Abbasi-Kolli M. Autophagy-related microRNAs: Possible regulatory roles and therapeutic potential in and gastrointestinal cancers. Pharmacol Res (2020) 161:105133. 10.1016/j.phrs.2020.105133 32822869

[5] Sanger HL Klotz G Riesner D Gross HJ Kleinschmidt AK. Viroids are single-stranded covalently closed circular RNA molecules existing as highly base-paired rod-like structures. Proc Natl Acad Sci (1976) 73:3852–56. 10.1073/pnas.73.11.3852 PMC431239 1069269

[6] Capel B Swain A Nicolis S Hacker A Walter M Koopman P. Circular transcripts of the testis-determining gene Sry in adult mouse testis. Cell (1993) 73:1019–30. 10.1016/0092-8674(93)90279-y 7684656

[7] Cocquerelle C Daubersies P Majerus MA Kerckaert JP Bailleul B. Splicing with inverted order of exons occurs proximal to large introns. EMBO J (1992) 11:1095–98. 10.1002/j.1460-2075.1992.tb05148.x PMC556550 1339341

[8] Cocquerelle C Mascrez B Hetuin D Bailleul B. Mis-splicing yields circular RNA molecules. FASEB J (1993) 7:155–60. 10.1096/fasebj.7.1.7678559 7678559

[9] Nigro JM Cho KR Fearon ER Kern SE Ruppert JM Oliner JD. Scrambled exons. Cell (1991) 64:607–13. 10.1016/0092-8674(91)90244-s 1991322

[10] Chen LL. The expanding regulatory mechanisms and cellular functions of circular RNAs. Nat Rev Mol Cell Biol (2020) 21:475–90. 10.1038/s41580-020-0243-y 32366901

[11] Zhang XO Dong R Zhang Y. Diverse alternative back-splicing and alternative splicing landscape of circular RNAs. Genome Res (2016) 26:1277–87. 10.1101/gr.202895.115 PMC5052039 27365365

[12] Zhang Y Zhang XO Chen T Xiang JF Yin QF Xing YH. Circular intronic long noncoding RNAs. Mol Cell (2013) 51:792–806. 10.1016/j.molcel.2013.08.017 24035497

[13] Li ZY Huang C Bao C Chen L Lin M Wang XL. Exon-intron circular RNAs regulate transcription in the nucleus. Nat Struct Mol Biol (2015) 22:256–64. 10.1038/nsmb.2959 25664725

[14] Jeck WR Sorrentino JA Wang K Slevin MK Burd CE Liu J. Circular RNAs are abundant, conserved, and associated with ALU repeats. RNA (2012) 19:141–57. 10.1261/rna.035667.112. PMC3543092 23249747

[15] Zhang XO Wang HB Zhang Y Lu XH Chen LL Yang L. Complementary sequence-mediated exon circularization. Cell (2014) 159:134–47. 10.1016/j.cell.2014.09.001 25242744

[16] Zhang Y Xue W Li X Zhang J Chen SY Zhang JL. The biogenesis of nascent circular RNAs. Cell Rep (2016) 15:611–24. 10.1016/j.celrep.2016.03.058 27068474

[17] Aufiero S Reckman YJ Pinto YM Creemers EE. Circular RNAs open a new chapter in cardiovascular biology. Nat Rev Cardiol (2019) 16:503–14. 10.1038/s41569-019-0185-2 30952956

[18] Liang D Wilusz JE. Short intronic repeat sequences facilitate circular RNA production. Genes Dev (2014) 28:2233–47. 10.1101/gad.251926.114 PMC4201285 25281217

[19] Li X Liu CX Xue W Zhang Y Jiang S Yin QF. Coordinated circRNA biogenesis and function with NF90/NF110 in viral infection. Mol Cell (2017) 67:214–27. 10.1016/j.molcel.2017.05.023 28625552

[20] Patino C Haenni AL Urcuqui-Inchima S. NF90 isoforms, a new family of cellular proteins involved in viral replication? Biochimie (2015) 108:20–4. 10.1016/j.biochi.2014.10.022 25447144

[21] Zheng XY Huang MG Xing L Yang R Wang XS Jiang R. The circRNA circSEPT9 mediated by E2F1 and EIF4A3 facilitates the carcinogenesis and development of triple-negative breast cancer. Mol Cancer (2020) 19:73. 10.1186/s12943-020-01183-9 32264877 PMC7137343

[22] Conn SJ Pillman KA Toubia J Conn VM Salmanidis M Phillips CA. The RNA binding protein quaking regulates formation of circRNAs. Cell (2015) 160:1125–34. 10.1016/j.cell.2015.02.014 25768908

[23] Kelly S Greenman C Cook PR Papantonis A. Exon skipping is correlated with exon circularization. J Mol Biol (2015) 427:2414–17. 10.1016/j.jmb.2015.02.018. 25728652

[24] Liang DM Tatomer DC Luo Z Wu H Yang L Chen LL. The output of protein-coding genes shifts to circular RNAs when the pre-mRNA processing machinery is limiting. Mol Cell (2017) 68:940–54. 10.1016/j.molcel.2017.10.034 PMC5728686 29174924

[25] Ashwal-Fluss R Meyer M Pamudurti NR Ivanov A Bartoket O Hanan M. CircRNA biogenesis competes with pre-mRNA splicing. Mol Cell (2014) 56:55–66. 10.1016/j.molcel.2014.08.019 25242144

[26] Kristensen LS Andersen MS Stagsted LVW Ebbesen KK Hansen TB Kjems J. The biogenesis, biology and characterization of circular RNAs. Nat Rev Genet (2019) 20:675–91. 10.1038/s41576-019-0158-7. 31395983

[27] Tabak HF Van der Horst G Smit J Winter AJ Mul Y Groot Koerkamp MJ. Discrimination between RNA circles, interlocked RNA circles and lariats using two-dimensional polyacrylamide gel electrophoresis. Nucleic Acids Res (1988) 16:6597–605. 10.1093/nar/16.14.6597 PMC338316 2456529

[28] Suzuki H Tsukahara T. A view of pre-mRNA splicing from RNase R resistant RNAs. Int J Mol Sci (2014) 15:9331–42. 10.3390/ijms15069331 PMC4100097 24865493

[29] Han YC Donovan J Rath S Whitney G Chitrakar A Korennykh A. Structure of human RNase L reveals the basis for regulated RNA decay in the IFN response. Science (2014) 343:1244–8. 10.1126/science.1249845. PMC4731867 24578532

[30] Park OH Ha H Lee YJ Boo SH Kwon DH Song HK. Endoribonucleolytic cleavage of m6A-containing RNAs by RNase P/MRP complex. Mol Cell (2019) 74:494–507. 10.1016/j.molcel.2019.02.034. 30930054

[31] Hansen TB Wiklund ED Bramsen JB Villadsen SB Statham AL Clark SJ. miRNA-dependent gene silencing involving Ago2-mediated cleavage of a circular antisense RNA. EMBO J (2011) 30:4414–22. 10.1038/emboj.2011.359 PMC3230379 21964070

[32] Kleaveland B Shi CY Stefano J. A network of noncoding regulatory RNAs acts in the mammalian brain. Cell (2018) 174:350–62. 10.1016/j.cell.2018.05.022 PMC6559361 29887379

[33] Pan ZQ Li GF Sun ML Xie L Liu D Zhang Q. MicroRNA-1224 splicing circularRNA-Filip1l in an Ago2-dependent manner regulates chronic inflammatory pain via targeting Ubr5. J Neurosci (2019) 39:2125–43. 10.1523/JNEUROSCI.1631-18.2018 PMC6507086 30651325

[34] Lasda E Parker R. Circular RNAs co-precipitate with extracellular vesicles: a possible mechanism for circRNA clearance. PloS One (2016) 11:e0148407. 10.1371/journal.pone.0148407 26848835 PMC4743949

[35] Enuka Y Lauriola M Feldman ME Sas-Chen A Ulitsky I Yarden Y. Circular RNAs are long-lived and display only minimal early alterations in response to a growth factor. Nucleic Acids Res (2015) 44:1370–83. 10.1093/nar/gkv1367 PMC4756822 26657629

[36] Huang C Liang DM Tatomer DC Wilusz JE. A length-dependent evolutionarily conserved pathway controls nuclear export of circular RNAs. Genes Dev (2018) 32:639–44. 10.1101/gad.314856.118. PMC6004072 29773557

[37] Gatfield D Hir HL Schmitt C Braun IC Köcher T Wilm M. The DExH/D box protein HEL/UAP56 is essential for mRNA nuclear export in Drosophila. Curr Biol (2001) 11:1716–21. 10.1016/s0960-9822(01)00532-2 11696332

[38] Roundtree IA Luo GZ Zhang ZJ Wang X Zhou T Cui YQ. YTHDC1 mediates nuclear export of N6-methyladenosine methylated mRNAs. Elife (2017) 6:e31311. 10.7554/eLife.31311 28984244 PMC5648532

[39] Chen RX Chen X Xia LP Zhang JX Pan ZZ Ma XD. N6-methyladenosine modification of circNSUN2 facilitates cytoplasmic export and stabilizes HMGA2 to promote colorectal liver metastasis. Nat Commun (2019) 10:4695. 10.1038/s41467-019-12651-2 31619685 PMC6795808

[40] Dong R Ma XK Chen LL Yang L. Increased complexity of circRNA expression during species evolution. RNA Biol (2016) 14:1064–74. 10.1080/15476286.2016.1269999 PMC5680680 27982734

[41] Lu TT Cui LL Zhou Y Zhu CR Fan DL Gong H. Transcriptome-wide investigation of circular RNAs in rice. RNA (2015) 21:2076–87. 10.1261/rna.052282.115 PMC4647462 26464523

[42] Broadbent KM Broadbent JC Ribacke U Wirth D Rinn JL Sabeti PC. Strand-specific RNA sequencing in plasmodium falciparum malaria identifies developmentally regulated long non-coding RNA and circular RNA. BMC Genomics (2015) 16:454. 10.1186/s12864-015-1603-4 26070627 PMC4465157

[43] Fan XY Zhang XN Wu XL Guo HS Hu YQ Tang FC. Single-cell RNA-seq transcriptome analysis of linear and circular RNAs in mouse preimplantation embryos. Genome Biol (2015) 16:148. 10.1186/s13059-015-0706-1 26201400 PMC4511241

[44] Memczak S Jens M Elefsinioti A Torti F Krueger J Rybak A. Circular RNAs are a large class of animal RNAs with regulatory potency. Nature (2013) 495:333–8. 10.1038/nature11928 23446348

[45] Wang PL Bao Y Yee MC Barrett SP Hogan GJ Olsen MN. Circular RNA is expressed across the eukaryotic tree of life. PloS One (2014) 9:e90859. 10.1371/journal.pone.0090859 24609083 PMC3946582

[46] Ji PF Wu WY Chen S Zheng Y Zhou L Zhang JY. Expanded expression landscape and prioritization of circular RNAs in mammals. Cell Rep (2019) 26:3444–60. 10.1016/j.celrep.2019.02.078 30893614

[47] Venø MT Hansen TB Venø ST Clausen BH Grebing M Finsen B. Spatio-temporal regulation of circular RNA expression during porcine embryonic brain development. Genome Biol (2015) 16:245. 10.1186/s13059-015-0801-3 26541409 PMC4635978

[48] Rybak-Wolf A Stottmeister C Glažar P Jens M Pino N Giusti S. Circular RNAs in the mammalian brain are highly abundant, conserved, and dynamically expressed. Mol Cell (2015) 58:870–85. 10.1016/j.molcel.2015.03.027. 25921068

[49] You XT Vlatkovic I Babic A Will T Epstein I Tushev G. Neural circular RNAs are derived from synaptic genes and regulated by development and plasticity. Nat Neurosci (2015) 18:603–10. 10.1038/nn.3975. PMC4376664 25714049

[50] Nicolet BP Engels S Aglialoro F Akker EVD Lindern MV Wolkers MC. Circular RNA expression in human hematopoietic cells is widespread and cell-type specific. Nucleic Acids Res (2018) 46:8168–80. 10.1093/nar/gky721 PMC6144802 30124921

[51] Salzman J Chen RE Olsen MN Wang PL Brown PO. Cell-type specific features of circular RNA expression. PloS Genet (2013) 9:e1003777. 10.1371/journal.pgen.1003777 24039610 PMC3764148

[52] Liu CX Li X Nan F Jiang S Gao X Guo SK. Structure and degradation of circular RNAs regulate PKR activation in innate immunity. Cell (2019) 177:865–80. 10.1016/j.cell.2019.03.046 31031002

[53] Wilusz JE. Circle the wagons: circular RNAs control innate immunity. Cell (2019) 177:797–99. 10.1016/j.cell.2019.04.020 PMC7112311 31051101

[54] Shi L Yan PJ Liang YL Sun Y Shen JL Zhou SJ. Circular RNA expression is suppressed by androgen receptor (AR)-regulated adenosine deaminase that acts on RNA (ADAR1) in human hepatocellular carcinoma. Cell Death Dis (2017) 8:e3171. 10.1038/cddis.2017.556 29144509 PMC5775411

[55] Shi YJ Fang N Li YD Guo ZZ Jiang W He YZ. circLPAR3 sponges miR-198 to facilitate esophageal cancer migration, invasion and metastasis. Cancer Sci (2020) 111:2824–36. 10.1111/cas.14511. PMC7419039 32495982

[56] Ogino S Chan AT Fuchs CS Giovannucci E. Molecular pathologic epidemiology of colorectal neoplasia: an emerging transdisciplinary and interdisciplinary field. Gut (2011) 60:397–411. 10.1136/gut.2010.217182. 21036793 PMC3040598

[57] Ogino S Nowak JA Hamada T Milner DA Nishihara R Jr. Insights into pathogenic interactions among environment, host, and tumor at the crossroads of molecular pathology and epidemiology. Annu Rev Pathol: Mechanisms Dis (2018) 14:83–103. 10.1146/annurev-pathmechdis-012418-012818 PMC6345592 30125150

[58] Li D Li ZQ Yang Y Zeng XY Li YP Du XG. Circular RNAs as biomarkers and therapeutic targets in environmental chemical exposure-related diseases. Environ Res (2019) 180:108825. 10.1016/j.envres.2019.108825 31683121

[59] Teran JC. Nutrition and liver diseases. Curr Gastroenterol Rep (1999) 1:335–340. 10.1007/s11894-999-0119-y 10980970

[60] Li PF Shan KS Liu Y Zhang Y Xu L Xu L. CircScd1 promotes fatty liver disease via the janus kinase 2/signal transducer and activator of transcription 5 pathway. Digest Dis Sci (2018) 64:113–22. 10.1007/s10620-018-5290-2 30259280

[61] Meinecke A Mitzka S Just A Cushman S Stojanović SD Xiao K. Cardiac endurance training alters plasma profiles of circular RNA MBOAT2. Am J Physiol Heart Circulatory Physiol (2020) 319:H13–21. 10.1152/ajpheart.00067.2020 32412780

[62] Chen DL Qi LK Guo YR Liu YD Tang XC Zhu XX. CircNF1-419 improves the gut microbiome structure and function in AD-like mice. Aging (Albany NY) (2020) 6(12):260–287. 10.18632/aging.102614 PMC6977659 31905172

[63] Hughes LAE Simons CCJM van den Brandt PA van Engeland M Weijenberg MP. Lifestyle, diet, and colorectal cancer risk according to (epi)genetic instability: current evidence and future directions of molecular pathological epidemiology. Curr Colorectal Cancer Rep (2017) 13(6):455–469. 10.1007/s11888-017-0395-0 29249914 PMC5725509

[64] Carr PR Alwers E Bienert S Weberpals J Kloor M Brenner H. Lifestyle factors and risk of sporadic colorectal cancer by microsatellite instability status: a systematic review and meta-analyses. Ann Oncol (2018) 29(4):825–834. 10.1093/annonc/mdy059 29438474

[65] Wang ST Cui WQ Pan D Jiang M Chang B Sang LX. Tea polyphenols and their chemopreventive and therapeutic effects on colorectal cancer. World J Gastroenterol (2020) 26(6):562–597. 10.3748/wjg.v26.i6.562 32103869 PMC7029350

[66] Zhu ZX Huang JG Li X Xing J Chen Q Liu RL. Gut microbiota regulate tumor metastasis via circRNA/miRNA networks. Gut Microbes (2020), 1–16. 10.1080/19490976.2020.1788891 PMC7524358 32686598

[67] Zhang HY Deng T Ge SH Liu Y Bai M Zhu KG. Exosome circRNA secreted from adipocytes promotes the growth of hepatocellular carcinoma by targeting deubiquitination-related USP7. Oncogene (2019) 38:2844–59. 10.1038/s41388-018-0619-z PMC6484761 30546088

[68] Shabaninejad Z Vafadar A Movahedpour A Ghasemi Y Namdar A Fathizadeh H. Circular RNAs in cancer: new insights into functions and implications in ovarian cancer. J Ovarian Res (2019) 12:84. 10.1186/s13048-019-0558-5 31481095 PMC6724287

[69] Nahand JS Jamshidi S Hamblin MR Mahjoubin-Tehran M Vosough M Jamali M. Circular RNAs: new epigenetic signatures in viral infections. Front Microbiol (2020) 11:1853. 10.3389/fmicb.2020.01853 32849445 PMC7412987

[70] Borran S Ahmadi G Rezaei S Anari MM Modabberi M Azarash Z. Circular RNAs: New players in thyroid cancer. Pathol - Res Pract (2020) 216:153217. 10.1016/j.prp.2020.153217 32987339

[71] Luo Z Rong ZY Zhang JM Zhu ZL Yu ZL Li TF. Circular RNA circCCDC9 acts as a miR-6792-3p sponge to suppress the progression of gastric cancer through regulating CAV1 expression. Mol Cancer (2020) 19:86. 10.1186/s12943-020-01203-8 32386516 PMC7210689

[72] Zhou C Liu HS Wang FW Hu T Liang ZX Lan N. CircCAMSAP1 promotes tumor growth in colorectal cancer via miR-328-5p/E2F1 axis. Mol Ther (2019) 28:914–28. 10.1016/j.ymthe.2019.12.008 PMC7054739 31951832

[73] Guo XF Zhou QB Su D Luo YM Fu ZQ Huang LY. Circular RNA circBFAR promotes the progression of pancreatic ductal adenocarcinoma via the miR-34b-5p/MET/Akt axis. Mol Cancer (2020) 19:83. 10.1186/s12943-020-01196-4 32375768 PMC7201986

[74] Yu J Xu QG Wang ZG Yang Y Zhang L Ma JZ. Circular RNA cSMARCA5 inhibits growth and metastasis in hepatocellular carcinoma. J Hepatol (2018) 68:1214–27. 10.1016/j.jhep.2018.01.012. 29378234

[75] Du WW Zhang C Yang WN Yong TQ Awan FM Yang BB. Identifying and characterizing circRNA-protein interaction. Theranostics (2017) 7:4183–91. 10.7150/thno.21299. PMC5695005 29158818

[76] Hu XT Wu DG He XT Zhao HY He ZH Lin JT. circGSK3β promotes metastasis in esophageal squamous cell carcinoma by augmenting β-catenin signling. Mol Cancer (2019) 18:160. 10.1186/s12943-019-1095-y 31722716 PMC6854808

[77] Yang HB Li XB Meng QT Sun H Wu SS Hu WW. CircPTK2 (hsa_circ_0005273) as a novel therapeutic target for metastatic colorectal cancer. Mol Cancer (2020) 19:13. 10.1186/s12943-020-1139-3 31973707 PMC6977296

[78] Chen YJ Yang F Fang EH Xiao WJ Mei H Li HH. Circular RNA circAGO2 drives cancer progression through facilitating HuR-repressed functions of AGO2-miRNA complexes. Cell Death Differ (2019) 26:1346–64. 10.1038/s41418-018-0220-6 PMC6748083 30341421

[79] Wang SH Zhang YJ Cai Q Ma MZ Jin LY Weng MZ. Circular RNA FOXP1 promotes tumor progression and Warburg effect in gallbladder cancer by regulating PKLR expression. Mol Cancer (2019) 18:145. 10.1186/s12943-019-1078-z 31623628 PMC6796492

[80] Jie MM Wu YR Gao MY Li XZ Liu C Ouyang Q. CircMRPS35 suppresses gastric cancer progression via recruiting KAT7 to govern histone modification. Mol Cancer (2020) 19:56. 10.1186/s12943-020-01160-2 32164722 PMC7066857

[81] Wang LY Long HY Zheng QH Bo XT Xiao XH Li B. Circular RNA circRHOT1 promotes hepatocellular carcinoma progression by initiation of NR2F6 expression. Mol Cancer (2019) 18:119. 10.1186/s12943-019-1046-7 31324186 PMC6639939

[82] Huang XC He M Huang S Lin RR Zhan M Yang D. Circular RNA circERBB2 promotes gallbladder cancer progression by regulating PA2G4-dependent rDNA transcription. Mol Cancer (2019) 18:166. 10.1186/s12943-019-1098-8 31752867 PMC6868820

[83] Ding LX Zhao YY Dang SW Wang Y Li XL Yu XT. Circular RNA circ-DONSON facilitates gastric cancer growth and invasion via NURF complex dependent activation of transcription factor SOX4. Mol Cancer (2019) 18:45. 10.1186/s12943-019-1006-2 30922402 PMC6437893

[84] Li XM Wang JJ Zhang C Lin C Zhang JM Zhang W. Circular RNA circITGA7 inhibits colorectal cancer growth and metastasis by modulating the Ras pathway and upregulating transcription of its host gene ITGA7. J Pathol (2018) 246:166–79. 10.1002/path.5125 29943828

[85] Liu BQ Yang GS Wang X Liu JF Lu ZH Wang Q. CircBACH1 (hsa_circ_0061395) promotes hepatocellular carcinoma growth by regulating p27 repression via HuR. J Cell Physiol (2020) 235:6929–41. 10.1002/jcp.29589 32003018

[86] Chen L Kong RJ Wu C Wang S Liu ZX Liu SP. Circ-MALAT1 functions as both an mRNA translation brake and a microRNA sponge to promote self-renewal of hepatocellular cancer stem cells. Adv Sci (2020) 7:1900949. 10.1002/advs.201900949 PMC7029649 32099751

[87] Wang Y Wang ZF. Efficient backsplicing produces translatable circular mRNAs. RNA (2015) 21:172–79. 10.1261/rna.048272.114 PMC4338345 25449546

[88] Yang Y Fan XJ Mao MW Song XW Wu P Zhang Y. Extensive translation of circular RNAs driven by N6-methyladenosine. Cell Res (2017) 27:626–41. 10.1038/cr.2017.31. PMC5520850 28281539

[89] Zheng X Chen LJ Zhou Y Wang Q Zheng ZJ Xu B. A novel protein encoded by a circular RNA circPPP1R12A promotes tumor pathogenesis and metastasis of colon cancer via Hippo-YAP signaling. Mol Cancer (2019) 18:47. 10.1186/s12943-019-1010-6 30925892 PMC6440158

[90] Pan ZH Cai JY Lin JT Zhou HN Peng JW Liang JL. A novel protein encoded by circFNDC3B inhibits tumor progression and EMT through regulating Snail in colon cancer. Mol Cancer (2020) 19:71. 10.1186/s12943-020-01179-5 32241279 PMC7114813

[91] Liang WC Wong CW Liang PP Shi M Cao Y Rao ST. Translation of the circular RNA circβ-catenin promotes liver cancer cell growth through activation of the Wnt pathway. Genome Biol (2019) 20:84. 10.1186/s13059-019-1685-4 31027518 PMC6486691

[92] Yang F Hu AP Li D Wang JQ Guo YH Liu Y. Circ-HuR suppresses HuR expression and gastric cancer progression by inhibiting CNBP transactivation. Mol Cancer (2019) 18:158. 10.1186/s12943-019-1094-z 31718709 PMC6852727

[93] Zhi XF Zhang JX Cheng ZY Bian LJ Qin J. circLgr4 drives colorectal tumorigenesis and invasion through Lgr4-targeting peptide. Int J Cancer (2019) 7:32549. 10.1002/ijc.32549 31269234

[94] An Y Cai HH Zhang Y Liu SY Duan YF Sun DL. circZMYM2 competed endogenously with miR-335-5p to regulate JMJD2C in pancreatic cancer. Cell Physiol Biochem (2018) 51:2224–36. 10.1159/000495868 30537731

[95] Guan Z Tan J Gao W Li X Yang YD Li XG. Circular RNA hsa_circ_0016788 regulates hepatocellular carcinoma tumorigenesis through miR-486/CDK4 pathway. J Cell Physiol (2018) 234:500–8. 10.1002/jcp.26612 29923236

[96] Xu Y Leng KM Yao Y Kang PC Liao GQ Han Y. A novel circular RNA, circ-CCAC1, contributes to CCA progression, induces angiogenesis, and disrupts vascular endothelial barriers. Hepatology (2020) 8:31493. 10.1002/hep.31493 32750152

[97] He YL Mingyan E Wang CB Liu GH Shi MR Liu S. CircVRK1 regulates tumor progression and radioresistance in esophageal squamous cell carcinoma by regulating miR-624-3p/PTEN/PI3K/AKT signaling pathway. Int J Biol Macromol (2019) 125:116–23. 10.1016/j.ijbiomac.2018.11.273 30508543

[98] Jian XY He H Zhu JH Zhang Q Zheng ZX Liang XJ. Hsa_circ_001680 affects the proliferation and migration of CRC and mediates its chemoresistance by regulating BMI1 through miR-340. Mol Cancer (2020) 19:20. 10.1186/s12943-020-1134-8 32005118 PMC6993513

[99] Cui CC Yang JB Li X Liu DL Fu LW Wang XW. Functions and mechanisms of circular RNAs in cancer radiotherapy and chemotherapy resistance. Mol Cancer (2020) 19:58. 10.1186/s12943-020-01180-y 32171304 PMC7071709

[100] Ma L Wang ZD Xie MY Quan YL Zhu WY Yang FM. Silencing of circRACGAP1 sensitizes gastric cancer cells to apatinib via modulating autophagy by targeting miR-3657 and ATG7. Cell Death Dis (2020) 11:169. 10.1038/s41419-020-2352-0 32139670 PMC7058073

[101] Peng L Sang HM Wei SC Li YY Jin DC Zhu XD. circCUL2 regulates gastric cancer malignant transformation and cisplatin resistance by modulating autophagy activation via miR-142-3p/ROCK2. Mol Cancer (2020) 19:156. 10.1186/s12943-020-01270-x 33153478 PMC7643398

[102] Li J Qin XP Wu RS Wan L Zhang L Liu R. Circular RNA circFBXO11 modulates hepatocellular carcinoma progress and oxaliplatin resistance through miR-605/FOXO3/ABCB1 axis. J Cell Mol Med (2020) 9:5152–61. 10.1111/jcmm.15162. PMC7205830 32222024

[103] Ou ZL Luo Z Wei W Liang S Gao TL Lu YB. Hypoxia-induced shedding of MICA and HIF1A-mediated immune escape of pancreatic cancer cells from NK cells: role of circ_0000977/miR-153 axis. RNA Biol (2019) 16:1592–603. 10.1080/15476286.2019.1649585 PMC6779391 31402756

[104] Huang XY Zhang PF Wei CY Peng R Lu JC Gao C. Circular RNA circMET drives immunosuppression and anti-PD1 therapy resistance in hepatocellular carcinoma via the miR-30-5p/snail/DPP4 axis. Mol Cancer (2020) 19:92. 10.1186/s12943-020-01213-6 32430013 PMC7236145

[105] Zhang PF Gao C Huang XY Lu JC Guo XJ Shi GM. Cancer cell-derived exosomal circUHRF1 induces natural killer cell exhaustion and may cause resistance to anti-PD1 therapy in hepatocellular carcinoma. Mol Cancer (2020) 19:110. 10.1186/s12943-020-01222-5 32593303 PMC7320583

[106] Wang X Li HJ Lu YJ Cheng LM. Circular RNAs in Human Cancer. Front Oncol (2021) 10:577118. 10.3389/fonc.2020.577118 33537235 PMC7848167

[107] Fang N Shi YJ Fan Y Long T Shu YQ Zhou JW. Circ_0072088 promotes proliferation, migration and invasion of esophageal squamous cell cancer by absorbing miR-377. J Oncol (2020) 2020:8967126. 10.1155/2020/8967126 33061973 PMC7542490

[108] Li J Li ZH Jiang P Peng MJ Zhang X Chen K. RNA IARS (circ-IARS) secreted by pancreatic cancer cells and located within exosomes regulates endothelial monolayer permeability to promote tumor metastasis. J Exp Clin Cancer Res (2018) 3:177. 10.1186/s13046-018-0822-3 PMC6069563 30064461

[109] Dai J Su YZ Zhong SY Cong L Liu B Yang JJ. Exosomes: key players in cancer and potential therapeutic strategy. Sig Transduct Target Ther (2020) 5:145. 10.1038/s41392-020-00261-0 PMC7406508 32759948

[110] Shang AQ Gu CZ Wang WW Wang X Sun JJ Zeng BJ. Exosomal circPACRGL promotes progression of colorectal cancer via the miR-142-3p/miR-506-3p-TGF-β1 axis. Mol Cancer (2020) 19:117. 10.1186/s12943-020-01235-0 32713345 PMC7384220

[111] Xie MY Yu T Jing XM Ma L Fan Y Yang FM. Exosomal circSHKBP1 promotes gastric cancer progression via regulating the miR-582-3p/HUR/VEGF axis and suppressing HSP90 degradation. Mol Cancer (2020) 19:112. 10.1186/s12943-020-01208-3 32600329 PMC7322843

[112] Liu DR Kang HX Gao MT Jin L Zhang F Chen DQ. Exosome-transmitted circ_MMP2 promotes hepatocellular carcinoma metastasis by upregulating MMP2. Mol Oncol (2020) 14:1365–80. 10.1002/1878-0261.12637 PMC7266270 31944556

[113] Wen GX Zhou T Gu WJ. The potential of using blood circluar RNA as liquid biopsy biomarker for human diseases. Protein Cell (2020) 10:1–368585. 10.1007/s13238-020-00799-3 PMC8674396 33131025

[114] Fan LY Cao Q Liu J Zhang JP Li BS. Circular RNA profiling and its potential for esophageal squamous cell cancer diagnosis and prognosis. Mol Cancer (2019) 18:16. 10.1186/s12943-018-0936-4 30674324 PMC6343327

[115] Chen W Quan YY Fan SY Wang H Liang JY Huang L. Exosome-transmitted circular RNA hsa_circ_0051443 suppresses hepatocellular carcinoma progression. Cancer Lett (2020) 475:119–28. 10.1016/j.canlet.2020.01.022 32014458

[116] Cheng XL Tian P Zheng WZ Yan XT. Piplartine attenuates the proliferation of hepatocellular carcinoma cells via regulating hsa_circ_100338 expression. Cancer Medcine (2020) 9:4265–73. 10.1002/cam4.3043. PMC7300402 32281302

[117] Gong YH Mao JZ Wu D Wang XM Li L Zhu L. Circ-ZEB1.33 promotes the proliferation of human HCC by sponging miR-200a-3p and upregulating CDK6. Cancer Cell Int (2018) 18:116. 10.1186/s12935-018-0602-3 30123094 PMC6090603

[118] Ye DX Wang SS Huang Y Chi P. A 3-circular RNA signature as a noninvasive biomarker for diagnosis of colorectal cancer. Cancer Cell Int (2019) 19:276. 10.1186/s12935-019-0995-7 31700498 PMC6829842

[119] Yang F Liu DY Guo JT Ge N Zhu P Liu X. Circular RNA circ-LDLRAD3 as a biomarker in diagnosis of pancreatic cancer. World J Gastroenterol (2017) 23:8345–54. 10.3748/wjg.v23.i47.8345 PMC5743505 29307994

[120] Jost I Shalamova LA Gerresheim GK Niepmann M Bindereif A Rossbach O. Functional sequestration of microRNA-122 from hepatitis C virus by circular RNA sponges. RNA Biol (2018) 15:1032–39. 10.1080/15476286.2018.1435248 PMC6161685 29486652

