# Supplementary material for: Research Progress of Circular RNA in Gastrointestinal Tumors
Source: Front Oncol. 2021 Apr 15;11:665246. doi: 10.3389/fonc.2021.665246 (PMC8082141; doi:10.3389/fonc.2021.665246)
Supplement: Supplementary file 1 [file Table_1.docx]

**Supplementary Table 1 Circular RNAs in esophageal squamous cell carcinoma (ESCC).**

| circRNAs | expression | mechanisms | target gene | function（promote +, suppress -) | Refs. |
| --- | --- | --- | --- | --- | --- |
| circLPAR3 | up | sponge  miR-198 | MET | migration(+), invasion(+). | [1] |
| circVRK1 | down | sponge  miR-624-3p | PTEN/PI3K/  AKT | proliferation (-), migration (-),  EMT (-), the sensitivity of ESCC cells to radiotherapy (+). | [2] |
| circ_0072088 | up | sponge  miR-377 | VEGF | [proliferation (+), migration (+),](http://www.chinapubmed.net/32495982)  [invasion(+).](http://www.chinapubmed.net/32495982) | [3] |
| has_circ_0006168 | up | sponge  miR-100 | mTOR | proliferation (+), migration (+),  invasion (+). | [4] |
| hsa_circ_0004771 | up | sponge  miR-339-5p | CDC25A | [proliferation (+), migration (+),](http://www.chinapubmed.net/32495982)  [invasion(+).](http://www.chinapubmed.net/32495982) | [5] |
| hsa_circ_0000654 | up | sponge  miR-149-5p | IL-6/STAT3 | [proliferation (+), migration (+),](http://www.chinapubmed.net/32495982)  [invasion(+).](http://www.chinapubmed.net/32495982) | [6] |
| circGSK3β | up | interact with GSK3β protein | Wnt/β-catenin | migration(+), invasion(+). | [7] |
| hsa_circ_0001946 | down | - | - | [proliferation (-), migration(-), invasion(-).](http://www.chinapubmed.net/32495982) | [8] |
| circ-SMAD7 | down | - | - | [proliferation (-), migration (-).](http://www.chinapubmed.net/32495982) | [9] |

## Supplementary Table 1 Reference

1. Shi YJ, Fang N, Li YD, [Guo](https://pubmed.ncbi.nlm.nih.gov/?term=Guo+Z&cauthor_id=32495982) ZZ, [Jiang](https://pubmed.ncbi.nlm.nih.gov/?term=Jiang+W&cauthor_id=32495982) W, [He](https://pubmed.ncbi.nlm.nih.gov/?term=He+Y&cauthor_id=32495982) YZ, et al. circLPAR3 sponges miR-198 to facilitate esophageal cancer migration, invasion and metastasis. *Cancer Sci*. (2020) 111: 2824-36. doi: 10.1111/cas.14511.
2. He YL, Mingyan E, Wang CB, Liu GH, Shi MR, Liu S. CircVRK1 regulates tumor progression and radioresistance in esophageal squamous cell carcinoma by regulating miR-624-3p/PTEN/PI3K/AKT signaling pathway. *International Journal of Biological Macromolecules*. (2019) 125: 116-23. doi:10.1016/ j.ijbiomac.2018.11.273.
3. [Fang](https://onlinelibrary.wiley.com/action/doSearch?ContribAuthorStored=Fang%2C+Na) N, [Shi](https://onlinelibrary.wiley.com/action/doSearch?ContribAuthorStored=Shi%2C+Yijun) YJ, Fan Y, Long T, Shu YQ, Zhou JW. Circ_0072088 promotes proliferation, migration and invasion of esophageal squamous cell cancer by absorbing miR-377. *Journal of Oncology.* (2020) 8967126. doi:org/10.1155/2020/ 8967126.
4. Shi YJ, Guo ZZ, Fang N, Jiang W, Fan Y, He YZ, et al. hsa_circ_0006168 sponges miR-100 and regulates mTOR to promote the proliferation, migration and invasion of esophageal squamous cell carcinoma. *Biomedicine & Pharmacotherapy*. (2019) 117: 109151. doi: 10.1016/j.biopha.2019.109151.
5. Huang EM, Fu JH, Yu QY, Xie PX, Yang ZX, Ji HL, et al. CircRNA hsa_circ_0004771 promotes esophageal squamous cell cancer progression via miR-339-5p/CDC25A axis. *Epigenomics*. (2020) 12: 587-603. doi: 10.2217/epi-2019-0404.
6. Xu ZQ, Tie XJ, Li N, Yi ZY, Shen FQ, Zhang Y. Circular RNA hsa_circ_0000654 promotes esophageal squamous cell carcinoma progression by regulating the miR‐149‐5p/IL‐6/STAT3 pathway. *UBMB Life*. (2020) 72: 426-39. doi: 10.1002/iub.2202.
7. Hu XT, Wu DG, He XT, Zhao HY, He ZH, Lin JT, et al. circGSK3β promotes metastasis in esophageal squamous cell carcinoma by augmenting β-catenin signling. *Mol Cancer*. (2019) 18: 160. doi: 10.1186/s12943-019-1095-y.
8. Fan LY, Cao Q, Liu J, Zhang JP, Li BS. Circular RNA profiling and its potential for esophageal squamous cell cancer diagnosis and prognosis. *Mol Cancer*. (2019) 18: 16. doi: 10.1186/s12943-018-0936-4.
9. Zhang YZ, Wang Q, Zhu DL, Rong J, Shi WH, Cao XF. Up-regulation of circ-SMAD7 inhibits tumor proliferation and migration in esophageal squamous cell carcinoma. *Biomedicine & Pharmacotherapy*. (2019) 111: 596-601. doi: 10.1016/j.biopha.2018.12.116.
