# Supplementary material for: Research Progress of Circular RNA in Gastrointestinal Tumors
Source: Front Oncol. 2021 Apr 15;11:665246. doi: 10.3389/fonc.2021.665246 (PMC8082141; doi:10.3389/fonc.2021.665246)
Supplement: Supplementary file 2 [file Table_2.docx]

**Supplement table 2 Circular RNAs in gastric cancer (GC).**

| circRNAs | expression | mechanisms | target gene | function（promote +, suppress -) | Refs. |
| --- | --- | --- | --- | --- | --- |
| circCCDC9 | down | sponge miR-6792-3p | CAV1 | proliferation (-), migration (-),  invasion(-) . | [1] |
| circRACGAP1 | up | sponge  miR-3657 | ATG7 | autophagy (+), sensitivity of GC cells to apatinib (+). | [2] |
| circCUL2 | down | sponge  miR-142-3p | ROCK2 | proliferation (-), migration (-), invasion(-), autophagy(-), cisplatin sensitivity (+). | [3] |
| circLMP2A | up | sponge  miR-3908 | TRIM59/  p53 | induce stemness in  EBV-associated gastric cancer. | [4] |
| hsa-circ-000684 | up | sponge  miR-186 | ZEB1 | proliferation (+), migration (+), invasion(+) , tube formation (+). | [5] |
| circ-CEP85L | down | sponge  miR-942-5p | NFKBIA | proliferation (-), migration (-),  invasion(-) . | [6] |
| circRHOBTB3 | down | sponge  miR-654-3p | p21 | proliferation (-), G1/S arrest (+). | [7] |
| circFN1 | up ( in CDDP-  resistant GC cells) | sponge  miR-182-5p | - | apoptosis(-), CDDP resistance(+). | [8] |
| circPIP5K1A | up | sponge  miR-671-5p | KRT80/PI3K/  AKT | proliferation (+), migration (+), invasion(+) , EMT (+). | [9] |
| has_circ_0006282 | up | sponge  miR-155 | FBXO22 | proliferation (+), migration (+), invasion(+) . | [10] |
| circPSMC3 | down | sponge  miR-296-5p | PTEN | proliferation (-), migration (-),  invasion(-) . | [11] |
| circPVT1 | up | sponge  miR-125 | E2F2 | proliferation (+). | [12] |
| circNHSL1 | up | sponge miR-1306-3p | SIX1/  vimentin | proliferation (+), migration (+),  invasion(+). | [13] |
| hsa_circ_0008035 | up | sponge  miR-599 | EIF4A1 | proliferation (+), apoptosis (-),  ferroptosis (-). | [14] |
| circMAT2B | up | sponge  miR-515-5p | HIF-1α | cell viability (+), proliferation (+), DNA  synthesis(+), glucose uptake (+),  lactate production (+). | [15] |
| circRNAs | expression | mechanisms | target gene | function（promote +, suppress -) | Refs. |
| circDUSP16 | up | sponge  miR-145-5p | IVNS1ABP | cell viability (+),  proliferation(+), invasion (+). | [16] |
| circHECTD1 | up | sponge  miR-1256 | β-catenin  /c-Myc | glutaminolysis (+), proliferation (+), migration (+) invasion (+). | [17] |
| hsa_circ_006100 | up | sponge  miR-195 | GPRC5A  /EGFR | proliferation (+), migration (+),  invasion(+). | [18] |
| circLARP4 | down | sponge  miR-424 | LAST1 | proliferation (-), invasion(-). | [19] |
| hsa_circ_0027599 | down | sponge miR-101-3p.1 | PHLAD1 | proliferation (-), migration (-),  invasion(-). | [20] |
| circYAP1 | down | sponge  miR-367-5p | p27 kip1 | proliferation (-), invasion(-). | [21] |
| circCACTIN | up | sponge  miR-331-3p | TGFBR1 | migration (+), invasion (+),  EMT (+). | [22] |
| circFAT1(e2) | down | sponge  miR-548g | YBX1 | proliferation (-), migration (-),  invasion(-). | [23] |
| circNRIP1 | up | sponge  miR-149-5p | AKT1/mTOR | migration (+), invasion (+). | [24] |
| circRBMS3 | up | sponge  miR-153 | SNAI1 | proliferation (+), invasion(+). | [25] |
| hsa_circ_0081143 | up | sponge  miR-646 | CDK6 | cisplatin resistance (+). | [26] |
| circOSBPL10 | up | sponge  miR-136-5p | WNT2 | proliferation (+), migration (+),  invasion(+). | [27] |
| circAKT3 | up | sponge  miR-198 | PIK3R1 | cisplatin resistance (+). | [28] |
| circDLST | up | sponge  miR-502-5p | NRAS/MEK1/  ERK1/2 | proliferation (+), DNA synthesis (+), invasion (+), liver metastasis (+). | [29] |
| circHIPK3 | up | sponge miR-653-5p/  miR-338-3p | NRP1 | proliferation (+), migration (+). | [30] |
| circSHKBP1 | up | sponge miR-582-3p and HSP90 | HUR/VEGF | proliferation (+), migration (+),  invasion (+), angiogenesis (+). | [31] |
| circAGO2 | up | sponge HuR | AGO2 | proliferation (+), migration (+),  invasion(+). | [32] |
| circHuR | down | sponge CNBP | HuR | proliferation (-), migration (-),  invasion(-). | [33] |
| circRNAs | expression | mechanisms | target gene | function（promote +, suppress -) | Refs. |
| circMRPS35 | down | act as a modular scaffold | KAT7 | [proliferation (-), invasion(-).](http://www.chinapubmed.net/32495982) | [34] |
| circDONSON | up | recruite the NURF complex | SOX4 | proliferation (+), migration (+),  invasion (+), apoptosis (-). | [35] |
| circ-CTNNB1 | up | bound DEAD-box polypeptide 3 (DDX3) | YY1/β-catenin | proliferation (+), invasion(+). | [36] |

## Supplementary Table 2 Reference

1. Luo Z, Rong ZY, Zhang JM, [Zhu](https://pubmed.ncbi.nlm.nih.gov/?term=Zhu+Z&cauthor_id=32386516) ZL, [Yu](https://pubmed.ncbi.nlm.nih.gov/?term=Yu+Z&cauthor_id=32386516) ZL, [Li](https://pubmed.ncbi.nlm.nih.gov/?term=Li+T&cauthor_id=32386516) TF, et al. Circular RNA circCCDC9 acts as a miR-6792-3p sponge to suppress the progression of gastric cancer through regulating CAV1 expression. *Mol Cancer*. (2020) 19: 86. doi: 10.1186/s12943-020-01203-8.
2. Ma L, Wang ZD, Xie MY, Quan YL, Zhu WY, Yang FM, et al. Silencing of circRACGAP1 sensitizes gastric cancer cells to apatinib via modulating autophagy by targeting miR-3657 and ATG7. *Cell Death Dis*. (2020) 11: 169. doi: 10.1038/s41419-020-2352-0.
3. Peng L, Sang HM, Wei SC, Li YY, Jin DC, Zhu XD, et al. circCUL2 regulates gastric cancer malignant transformation and cisplatin resistance by modulating autophagy activation via miR-142-3p/ROCK2. *Mol Cancer*. (2020) 19: 156. doi: 10.1186/s12943-020-01270-x.
4. Gong LP, Chen JN, Dong M, Xiao ZD, Feng ZY, Pan YH, et al. Epstein-Barr virus‐derived circular RNA LMP2A induces stemness in EBV‐associated gastric cancer. *EMBO Reports*. (2020) 21: e49689. doi: 10.15252/embr.201949689.
5. Lin S, Song SZ, Sun R, Zhang MB, Du YT, Zhang DD, et al. Oncogenic circular RNA Has-circ-000684 interacts with microRNA‐186 to upregulate ZEB1 in gastric cancer. *The FASEB Journal*. (2020) 34: 8187-203. doi: 10.1096/fj.201903246R.
6. Lu J, Wang YH, Huang XY, Xie JW, Wang JB, Lin JX, et al. circ-CEP85L suppresses the proliferation and invasion of gastric cancer by regulating NFKBIA expression via miR‐942‐5p. *Journal of Cellular Physiology*. (2020) 235: 6287-99. doi: 10.1002/jcp.29556.
7. Deng GX, Mou TY, He JY, Chen D, Lv DJ, Liu H, et al. Circular RNA circRHOBTB3 acts as a sponge for miR-654-3p inhibiting gastric cancer growth. *Journal of Experimental & Clinical Cancer Research.* (2020) 39: 1. doi: 10.1186/s13046-019-1487-2.
8. Huang XX, Zhang Q, Hu H, Jin Y, Zeng AL, Xia YB, et al. A novel circular RNA circFN1 enhances cisplatin resistance in gastric cancer via sponging miR‐182‐5p. *Journal of Cellular Biochemistry*. (2020) 1: 29641. doi: 10.1002/jcb.29641.
9. Song H, Xu YX, Xu T, Fan RZ, Jiang T, Cao M, et al. CircPIP5K1A activates KRT80 and PI3K/AKT pathway to promote gastric cancer development through sponging miR-671-5p. *Biomedicine & Pharmacotherapy*. (2020) 126: 109941. doi: 10.1016/j.biopha.2020.109941.
10. He YR, Wang, YF, Liu L, Liu SJ, Liang LC, Chen YN, et al. Circular RNA circ_0006282 contributes to the progression of gastric cancer by sponging miR-155 to upregulate the expression of FBXO22. *Onco Targets Ther*. 2020; 13: 1001-10. doi: 10.2147/OTT.S228216.
11. Rong DW, Lu C, Zhang B, Fu K, Zhao SL, Tang WW, et al. CircPSMC3 suppresses the proliferation and metastasis of gastric cancer by acting as a competitive endogenous RNA through sponging miR-296-5p. *Mol Cancer*. (2019) 18: 25. doi: 10.1186/s12943-019-0958-6.
12. Chen J, Li Y, Zheng QP, Bao CY, He J, Chen B, et al. Circular RNA profile identifies circPVT1 as a proliferative factor and prognostic marker in gastric cancer. *Cancer Letters*. (2017) 388: 208-19. doi: 10.1016/j.canlet.2016.12.006.
13. Zhu ZL, Rong ZY, Luo Z, Yu ZL, Zhang J, Qiu ZJ, et al. Circular RNA circNHSL1 promotes gastric cancer progression through the miR-1306-3p/SIX1/vimentin axis. *Mol Cancer*. (2019) 18: 126. doi: 10.1186/s12943-019-1054-7.
14. Li C, Tian Y, Liang Y, Li QC. Circ_0008035 contributes to cell proliferation

and inhibits apoptosis and ferroptosis in gastric cancer via miR-599/EIF4A1 axis. *Cancer Cell Int*. (2020) 20: 84. doi: 10.1186/s12935-020-01168-0.

1. Liu J, Liu HY, Zeng QS, Xu P, Liu MX, Yang N. Circular RNA circ-MAT2B facilitates glycolysis and growth of gastric cancer through regulating the miR-515-5p/HIF-1α axis. *Cancer Cell Int*. (2020) 20: 171. doi: 10.1186/s12935-020-01256-1.
2. Zhang ZZ, Wang CJ, Zhang YQ, Yu ST, Zhao G, Xu J. CircDUSP16 promotes the tumorigenesis and invasion of gastric cancer by sponging miR-145-5p.*Gastric Cancer*. (2020) 23: 437-48. doi: 10.1007/s10120-019-01018-7.
3. Cai J, Chen ZQ, Wang JG, Wang JF, Chen XJ, Liang LH, et al. circHECTD1 facilitates glutaminolysis to promote gastric cancer progression by targeting miR-1256 and activating β-catenin/c-Myc signaling. *Cell Death Dis*. (2019) 10: 576. doi: 10.1038/s41419-019-1814-8.
4. Liang M, Huang GQ, Liu ZY, Wang Q, Yu ZJ, Liu ZL, et al. Elevated levels of hsa_circ_006100 in gastric cancer promote cell growth and metastasis via miR‐195/GPRC5A signalling. *Cell Proliferation*. (2019) 52: e12661. doi: 10.1111/cpr.12661.
5. Zhang J, Liu H, Hou LD, Wang G, Zhang R, Huang YX, et al. Circular RNA_LARP4 inhibits cell proliferation and invasion of gastric cancer by sponging miR-424-5p and regulating LATS1 expression. *Mol Cancer*. (2017) 16: 151. doi: 10.1186/s12943-017-0719-3.
6. Wang L, Shen JY, Jiang YS. Circ_0027599/PHDLA1 suppresses gastric cancer progression by sponging miR-101-3p.1. *Cell Biosci*. (2018) 8: 58. doi: 10.1186/s13578-018-0252-0.
7. Liu H, Liu Y, Bian ZL, Zhang J, Zhang R, Chen XY, et al. Circular RNA YAP1 inhibits the proliferation and invasion of gastric cancer cells by regulating the miR-367-5p/p27 Kip1 axis. *Mol Cancer*. (2018) 17: 151. doi: 10.1186/s12943-018-0902-1.
8. Zhang L, Song X, Chen X, Wang Q, Zheng X, Wu CP, et al. Circular RNA circCACTIN promotes gastric cancer progression by sponging miR-331-3p and regulating TGFBR1 expression. *International Journal of Biological Sciences*. (2019) 15: 1091-103. doi: 10.7150/ijbs.31533.
9. Fang J, Hong H, Xue XF, Zhu XG, Jiang LH, Qin MD, et al. A novel circular RNA, circFAT1(e2), inhibits gastric cancer progression by targeting miR-548g in the cytoplasm and interacting with YBX1 in the nucleus. *Cancer Lett*. 2018; 442: 222-32. doi: 10.1016/j.canlet.2018.10.040.
10. Zhang X, Wang S, Wang HX, Cao JC, Huang XX, Chen Z, et al. Circular RNA circNRIP1 acts as a microRNA-149-5p sponge to promote gastric cancer progression via the AKT1/mTOR pathway. *Mol Cancer*. (2019) 18: 20. doi: 10.1186/s12943-018-0935-5.
11. Li GY, Xue MH, Yang F, Jin YH, Fan YY, Li W. CircRBMS3 promotes gastric cancer tumorigenesis by regulating miR-153-SNAI1 axis. *Journal of Cellular Physiology*. (2018) 234: 3020-8. doi: 10.1002/jcp.27122.
12. Xue MH, Li GY, Fang XJ, Wang LL, Jin YH, Zhou QL. hsa_circ_0081143

promotes cisplatin resistance in gastric cancer by targeting miR-646/CDK6 pathway. *Cancer Cell Int*. (2019) 19: 25. doi: 10.1186/s12935-019-0737-x.

1. Wang S, Zhang X, Li Z, Wang WZ, Li BW, Huang XX, et al. Circular RNA profile identifies circOSBPL10 as an oncogenic factor and prognostic marker in gastric cancer. *Oncogene*. (2019) 38: 6985-7001. doi: 10.1038/s41388-019-0933- 0.
2. Huang XX, Li Z, Zhang Q, Wang WZ, Li BW, Wang L, et al. Circular RNA AKT3 upregulates PIK3R1 to enhance cisplatin resistance in gastric cancer via miR-198 suppression. *Mol Cancer*. (2019) 18: 71. doi: 10.1186/s12943-019-0969 -3.
3. Zhang J, Hou LD, Liang R, Chen XY, Zhang R, Chen W, et al. CircDLST promotes the tumorigenesis and metastasis of gastric cancer by sponging miR-502-5p and activating the NRAS/MEK1/ERK1/2 signaling. *Mol Cancer*. (2019) 18: 80. doi: 10.1186/s12943-019-1015-1.
4. Jin Y, Che XF, Qu XJ, Li X, Lu WQ, Wu J, et al. CircHIPK3 promotes metastasis of gastric cancer via miR-653-5p/miR-338-3p-NRP1 axis under a long-term hypoxic microenvironment. *Front Oncol.* (2020) 10: 1612. doi: 10.3389/fonc.2020.01612.
5. Xie MY, Yu T, Jing XM, Ma L, Fan Y, Yang FM, et al. Exosomal circSHKBP1 promotes gastric cancer progression via regulating the miR-582-3p/HUR/VEGF axis and suppressing HSP90 degradation. *Mol Cancer*. (2020) 19: 112. doi: 10.1186/s12943-020-01208-3.
6. Chen YJ, Yang F, Fang EH, Xiao WJ, Mei H, Li HH, et al. Circular RNA circAGO2 drives cancer progression through facilitating HuR-repressed functions of AGO2-miRNA complexes. *Cell Death & Differentiation*. (2019) 26: 1346-64. doi: 10.1038/s41418-018-0220-6.
7. Yang F, Hu AP, Li D, Wang JQ, Guo YH, Liu Y, et al. Circ-HuR suppresses HuR expression and gastric cancer progression by inhibiting CNBP transactivation. *Mol Cancer*. (2019) 18: 158. doi: 10.1186/s12943-019-1094-z.
8. Jie MM, Wu YR, Gao MY, Li XZ, Liu C, Ouyang Q, et al. CircMRPS35 suppresses gastric cancer progression via recruiting KAT7 to govern histone modification. *Mol Cancer*. (2020) 19: 56. doi: 10.1186/s12943-020-01160-2.
9. Ding LX, Zhao YY, Dang SW, Wang Y, Li XL, Yu XT, et al. Circular RNA circ-DONSON facilitates gastric cancer growth and invasion via NURF complex dependent activation of transcription factor SOX4. *Mol Cancer*. (2019) 18: 45. doi: 10.1186/s12943-019-1006-2.
10. Yang F, Fang EH, Mei H, Chen YJ, Li HH, Li D, et al. Cis-acting circ-CTNNB1 promotes β-catenin signaling and cancer progression via DDX3-mediated transactivation of YY1. *Cancer Research*. (2018) 79: 557-71. doi: 10.1158/0008-5472.CAN-18-1559.
