# Supplementary material for: Research Progress of Circular RNA in Gastrointestinal Tumors
Source: Front Oncol. 2021 Apr 15;11:665246. doi: 10.3389/fonc.2021.665246 (PMC8082141; doi:10.3389/fonc.2021.665246)
Supplement: Supplementary file 3 [file Table_3.docx]

**Supplementary Table 3 Circular RNAs in colorectal cancer (CRC).**

| circRNAs | expression | mechanisms | target gene | function（promote +, suppress -) | Refs. |
| --- | --- | --- | --- | --- | --- |
| circCAMSAP1 | up | sponge  miR-328-5p | E2F1 | proliferation (+). | [1] |
| circITGA7 | down | sponge  miR-370-3p | ITGA7 | proliferation (-), migration (-), invasion (-). | [2] |
| hsa_circ_001680 | up | sponge  miR-340 | BMI1 | proliferation (+), migration (+),  irinotecan chemoresistance (+). | [3] |
| circPAGRAL | up (in CRC cells treated with tumor-derived exosomes) | sponge  miR-142-3p /miR-506-3p | TGF-β1 | proliferation (+), migration (+), invasion(+). | [4] |
| hsa_circ_0079662 | up (in drug-resistant  cell lines) | sponge  miR-324-5p | HOXA9/  TNF-α | oxaliplatin resistance (+). | [5] |
| circ5615 | up | sponge  miR-149-5p | β-catenin/  cyclin1 | proliferation (+),  cell cycle acceleration (+). | [6] |
| hsa_circ_0004277 | up | sponge  miR-512-5p | PTMA | proliferation (+), apoptosis (-). | [7] |
| hsa_circ_0005963 | up (in oxaliplatin-  resistant cells) | sponge  miR-122 | PKM2 | glycolysis (+),  chemoresistance (+). | [8] |
| circHUWE1 | up | sponge  miR-486 | - | proliferation (+), migration (+), invasion(+). | [9] |
| hsa_circ_0137008 | down | sponge  miR-338-5p | EMT | migration (-), invasion (-),  EMT (-). | [10] |
| circVAPA | up | sponge  miR-125a | CREB5 | cells cycle progression (+),  migration (+), invasion (+),  glycolysis (+). | [11] |
| circ_0060745 | up | sponge  miR-4736 | CSE1L | proliferation (+), migration (+), invasion(+). | [12] |
| circ_0007142 | up | sponge  miR-122-5p | CDC25A | proliferation (+), migration (+), invasion(+). | [13] |
| circFARSA | up | sponge  miR-330-5p | LASP1 | proliferation (+), migration (+), invasion(+). | [14] |
| circRUNX1 | up | sponge  miR-145-5p | IGF1 | proliferation (+), migration (+), apoptosis(-). | [15] |
| circRNAs | expression | mechanisms | target gene | function（promote +, suppress -) | Refs. |
| circ_0021977 | down | sponge  miR-10b-5p | P21/P53 | proliferation (-), migration (-), invasion(-). | [16] |
| hsa_circ_0001178 | up | sponge  miR-382/miR-587/  miR-616 | ZEB1 | migration(+), invasion(+). | [17] |
| circIFT80 | up | sponge  miR-1236-3p | HOXB7 | proliferation (+), migration (+), invasion(+). | [18] |
| hsa_circ_0136666 | up | sponge  miR-136 | SH2B1 | proliferation (+), migration (+), invasion(+). | [19] |
| hsa_circ_0009361 | down | sponge  miR-582 | APC2 | proliferation (-), migration (-), invasion (-), EMT (-). | [20] |
| hsa_circ_101555 | up | sponge  miR-597-5p | CDK6, RPA3 | proliferation (+), apoptosis (-). | [21] |
| hsa_circ_0071589 | up | sponge  miR-600 | EZH2 | proliferation (+), migration (+), invasion(+). | [22] |
| circHIPK3 | up | sponge  miR-7 | FAK, IGF1R, EGFR, YY1 | proliferation (+), migration (+), invasion (+), apoptosis(-). | [23] |
| circPTK2 | up | bind to vimentin protein | - | proliferation (+), migration (+), invasion(+)，EMT(+). | [24] |
| circNSUN2 | up | N6-methyladenosine modification | circNSUN2/  IGF2BP2/  HMGA2 RNA-protein ternary complex | export to the cytoplasm (+)，  liver metastasis (+). | [25] |
| circPPP1R12A | up | encode  circPPP1R12A-73aa | hippo-YAP | proliferation (+), migration (+), invasion (+). | [26] |
| circFNDC3B | down | encode  circFNDC3B-218aa | Snail, FBP1 | proliferation (-), migration (-), invasion (-). | [27] |
| circLgr4 | up | encode peptide | Lgr4/Wnt/  β-catenin | colorectal CSC self-renewal(+)，proliferation (+)，invasion(+). | [28] |
| hsa_circ_0044556 | up | - | - | proliferation (+), migration (+), invasion(+). | [29] |
| circCCDC66 | up | - | - | proliferation (+), migration (+), invasion (+). | [30] |

## Supplementary Table 3 Reference

1. Zhou C, Liu HS, Wang FW, Hu T, Liang ZX, Lan N, et al. CircCAMSAP1 promotes tumor growth in colorectal cancer via miR-328-5p/E2F1 axis. *Molecular Therapy*. (2019) 28: 914-28. doi: 10.1016/j.ymthe.2019.12.008.
2. Li XM, Wang JJ, Zhang C, Lin C, Zhang JM, Zhang W, et al. Circular RNA circITGA7 inhibits colorectal cancer growth and metastasis by modulating the Ras pathway and upregulating transcription of its host gene ITGA7. *The Journal of Pathology*. (2018) 246: 166-79. doi: 10.1002/path.5125.
3. Jian XY, He H, Zhu JH, Zhang Q, Zheng ZX, Liang XJ, et al. Hsa_circ_001680 affects the proliferation and migration of CRC and mediates its chemoresistance by regulating BMI1 through miR-340. *Mol Cancer*. (2020) 19: 20. doi: 10.1186/s12943-020-1134-8.
4. Shang AQ, Gu CZ, Wang WW, Wang X, Sun JJ, Zeng BJ, et al. Exosomal circPACRGL promotes progression of colorectal cancer via the miR-142-3p/miR-506-3p-TGF-β1 axis. *Mol Cancer*. (2020) 19: 117. doi: 10.1186/s12943-020-01235-0.
5. Lai MF, Liu GJ, Li RJ, Bai H, Zhao JZ, Xiao P, et al. Hsa_circ_0079662 induces the resistance mechanism of the chemotherapy drug oxaliplatin through the TNF-α pathway in human colon cancer. *Journal of Cellular and Molecular Medicine*. (2020) 24: 5021-27. doi: 10.1111/jcmm.15122.
6. Ma ZF, Han CC, Xia WJ, Wang SW, Li X, Fang PQ, et al. circ5615 functions as a ceRNA to promote colorectal cancer progression by upregulating TNKS. *Cell Death Dis*. (2020) 11:356.  doi: 10.1038/s41419-020-2514-0.
7. Yang L, Sun HW, Liu X, Chen JX, Tian ZM, Xu J, et al. Circular RNA hsa_circ_0004277 contributes to malignant phenotype of colorectal cancer by sponging miR‐512‐5p to upregulate the expression of PTMA. [*J Cell Physiol*](http://www.chinapubmed.net/search/?f_journal=J%20Cell%20Physiol)*.* (2020): 29484. doi: 10.1002/jcp.29484.
8. [Wang](https://pubmed.ncbi.nlm.nih.gov/?term=Wang+X&cauthor_id=31901148) [XY](https://pubmed.ncbi.nlm.nih.gov/31901148/#affiliation-1), [Zhang](https://pubmed.ncbi.nlm.nih.gov/?term=Zhang+H&cauthor_id=31901148) [HY](https://pubmed.ncbi.nlm.nih.gov/31901148/#affiliation-1), [Yang](https://pubmed.ncbi.nlm.nih.gov/?term=Yang+H&cauthor_id=31901148) HO, Bai M, Ning T, Deng T, et al. Exosome-delivered circRNA promotes glycolysis to induce chemoresistance through the miR-122-PKM2 axis in colorectal cancer. *Mol Oncol*. (2020) 14: 539-55. doi: 10.1002/1878-0261.12629.
9. Chen HY, Li XN, Ye CX, Chen ZL, Wang ZJ. Circular RNA circHUWE1 is

upregulated and promotes cell proliferation, migration and invasion in colorectal cancer by sponging miR-486. *Onco Targets Ther*. (2020) 13: 423-34. doi: 10.2147/OTT.S233338.

1. Yang ZF, Zhang JJ, Lu DH, Sun Y, Zhao XY, Wang XQ, et al. Hsa_circ_0137008 suppresses the malignant phenotype in colorectal cancer by acting as a microRNA-338-5p sponge. *Cancer Cell Int*. (2020) 20: 67. doi: 10.1186/s12935-020-1150-1.
2. Zhang XY, Xu YY, Yamaguchi KJ, Hu JP, Zhang LB, Wang JF, et al. Circular RNA circVAPA knockdown suppresses colorectal cancer cell growth process by regulating miR-125a/CREB5 axis. *Cancer Cell Int*. (2020) 20: 103. doi: 10.1186/s12935-020-01178-y.
3. Wang XB, Ren YY, Ma SY, Wang SY. Circular RNA 0060745, a novel circRNA, promotes colorectal cancer cell proliferation and metastasis through miR-4736 sponging. *Onco Targets Ther*. (2020) 13: 1941-51. doi: 10.2147/OTT.S240642.
4. Yin WZ, Xu J, Li C, Dai XK, Wu T, Wen JF. Circular RNA circ_0007142 facilitates colorectal cancer progression by modulating CDC25A expression via miR-122-5p.*Onco Targets Ther.* (2020) 13:3689-701. doi:10.2147/TTT.S238338.
5. Lu CX, Fu L, Qian XY, Dou LS, Cang SD. Knockdown of circular RNA circ-FARSA restricts colorectal cancer cell growth through regulation of miR-330-5p/LASP1 axis. *Archives of Biochemistry and Biophysics*. (2020) 689: 108434. doi: 10.1016/j.abb.2020.108434.
6. Chen ZL, Li XN, Ye CX, Chen HY, Wang ZJ. Elevated levels of circRUNX1 in colorectal cancer promote cell growth and metastasis via miR-145-5p/IGF1 signalling. *Onco Targets Ther*. (2020) 13: 4035-48. doi: 10.2147/OTT.S254133.
7. Lu C, Jiang W, Hui BQ, Rong DW, Fu K, Dong CX, et al. The circ_0021977/miR‐10b‐5p/P21 and P53 regulatory axis suppresses proliferation, migration, and invasion in colorectal cancer. *Journal of Cellular Physiology*. (2020) 235: 2273-85. doi: 10.1002/jcp.29135.
8. Ren CF, Zhang ZM, Wang SH, Zhu WT, Zheng PG, Wang WH. Circular RNA hsa_circ_0001178 facilitates the invasion and metastasis of colorectal cancer through upregulating ZEB1 via sponging multiple miRNAs. *Biological Chemistry*. (2020) 401: 487-96. doi: 10.1515/hsz-2019-0350.
9. Feng WM, Gong H, Wang YC, Zhu GL, Xue T, Wang Y, et al. circIFT80 functions as a ceRNA of miR-1236-3p to promote colorectal cancer progression. *Mol Ther Nucleic Acids*. (2019) 18: 375-87. doi: 10.1016/j.omtn.2019.08.024.
10. Jin CH, Wang AH, Liu LB, Wang GP, Li GS. Hsa_circ_0136666 promotes the proliferation and invasion of colorectal cancer through miR-136/SH2B1 axis. *Journal of Cellular Physiology*. (2019) 234: 7247-56. doi: 10.1002/jcp.27482.
11. Geng YT, Zheng X, Hu WW, Wang Q, Xu YJ, He WT, et al. Hsa_circ_0009361 acts as the sponge of miR-582 to suppress colorectal cancer progression by regulating APC2 expression. *Clinical Science*. (2019) 133: 1197-213. doi: 10.1042/CS20190286.
12. Chen ZL, Ren R, Wan DW, Wang YL, Xue XF, Jiang M, et al. Hsa_circ_101555 functions as a competing endogenous RNA of miR-597-5p to promote colorectal cancer progression. *Oncogene*. (2019) 38: 6017-34. doi:10.1038/s41388-019- 0857-8.
13. [Wang Y](https://pubmed.ncbi.nlm.nih.gov/?term=Yong+W&cauthor_id=29710537), [Xuan ZQ](https://pubmed.ncbi.nlm.nih.gov/?term=Zhuoqi+X&cauthor_id=29710537), [Wang BC](https://pubmed.ncbi.nlm.nih.gov/?term=Baocheng+W&cauthor_id=29710537), Zhang DS, Zhang C, Sun YM. Hsa_circ_0071589 promotes carcinogenesis via the miR-600/EZH2 axis in colorectal cancer. *Biomedicine & Pharmacotherapy*.(2018)102:1188-94. doi:10.1016/j.biopha.2018.

03.085.

1. Zeng KX, Chen XX, Xu M, Liu XX, Hu XX, Xu T, et al. CircHIPK3 promotes colorectal cancer growth and metastasis by sponging miR-7. *Cell Death Dis*. (2018) 9: 417. doi: 10.1038/s41419-018-0454-8.
2. Yang HB, Li XB, Meng QT, Sun H, Wu SS, Hu WW, et al. CircPTK2 (hsa_circ_0005273) as a novel therapeutic target for metastatic colorectal cancer. *Mol Cancer*. (2020) 19: 13. doi: 10.1186/s12943-020-1139-3.
3. Chen RX, Chen X, Xia LP, Zhang JX, Pan ZZ, Ma XD, et al. N6-methyladenosine modification of circNSUN2 facilitates cytoplasmic export and stabilizes HMGA2 to promote colorectal liver metastasis. *Nat Commun.* (2019) 10: 4695. doi: 10.1038/s41467-019-12651-2.
4. Zheng X, Chen LJ, Zhou Y, Wang Q, Zheng ZJ, Xu B, et al. A novel protein encoded by a circular RNA circPPP1R12A promotes tumor pathogenesis and metastasis of colon cancer via Hippo-YAP signaling. *Mol Cancer*. (2019) 18: 47. doi: 10.1186/s12943-019-1010-6.
5. Pan ZH, Cai JY, Lin JT, Zhou HN, Peng JW, Liang JL, et al. A novel protein encoded by circFNDC3B inhibits tumor progression and EMT through regulating Snail in colon cancer. *Mol Cancer*. (2020) 19: 71. doi: 10.1186/s12943-020-01179-5.
6. Zhi XF, Zhang JX, Cheng ZY, Bian LJ, Qin J. circLgr4 drives colorectal tumorigenesis and invasion through Lgr4‐targeting peptide. *International Journal of Cancer.* (2019) 7: 32549. doi: 10.1002/ijc.32549.
7. Jing L, Wu JH, Tang XC, Ma M, Long F, Tian BN, et al. Identification of circular RNA hsa_circ_0044556 and its effect on the progression of colorectal cancer. *Cancer Cell International*. (2020) 20: 427. doi:10.1186/s12935-020- 01523-1.
8. Hsiao KY, Lin YC, Gupta SK, Chang N, Yen LS, Sun HS, et al. Noncoding effects of circular RNA CCDC66 promote colon cancer growth and metastasis. *Cancer Research*. (2017) 77: 2339-50. doi: 10.1158/0008-5472.CAN-16-1883.
