# Supplementary material for: Research Progress of Circular RNA in Gastrointestinal Tumors
Source: Front Oncol. 2021 Apr 15;11:665246. doi: 10.3389/fonc.2021.665246 (PMC8082141; doi:10.3389/fonc.2021.665246)
Supplement: Supplementary file 4 [file Table_4.docx]

**Supplementary Table 4 Circular RNAs in pancreatic cancer.**

| circRNAs | expression | mechanisms | target gene | function（promote +, suppress -) | Refs. |
| --- | --- | --- | --- | --- | --- |
| circBFAR | up | sponge miR-34b-5p | MET / PI3K / Akt | proliferation (+), migration (+),  invasion (+). | [1] |
| circZMYM2 | up | sponge miR-335-5p | JMJD2C | proliferation (+), migration (+),  invasion (+)， apoptosis (-). | [2] |
| circ_0000977 | up | sponge miR-153 | HIF1A/  ADAM10 | immune escape. | [3] |
| circIARS | up | sponge miR-122 | ZO-1/RhoA/RhoA-GTP/  F-action | endothelial monolayer permeability (+), invasion (+), metastasis (+). | [4] |
| hsa_circ_001653 | up | sponge miR-377 | HOXC6 | cell viability (+), cell cycle (+),  invasion (+), apoptosis (-). | [5] |
| circ_0007534 | up | sponge miR-625 and miR-892b | Bcl-2/  caspase-3 | proliferation (+), migration (+),  invasion (+), apoptosis (-). | [6] |
| circLDLRAD3 | up | sponge miR-137-3p | PTN | proliferation (+), migration (+),  invasion (+). | [7] |
| circRNA_000864 | down | sponge  miR-361-3p | BTG2 | cell viability (-), cell cycle (-),  invasion (-), apoptosis (+). | [8] |

**Supplementary Table 4 Reference**

1. Guo XF, Zhou QB, Su D, Luo YM, Fu ZQ, Huang LY, et al. Circular RNA circBFAR promotes the progression of pancreatic ductal adenocarcinoma via the miR-34b-5p/MET/Akt axis. *Mol Cancer*. (2020) 19: 83. doi: 10.1186/s12943-020-01196-4.
2. An Y, Cai HH, Zhang Y, Liu SY, Duan YF, Sun DL, et al. circZMYM2 competed endogenously with miR-335-5p to regulate JMJD2C in pancreatic cancer. *Cellular Physiology and Biochemistry*. (2018) 51: 2224-36. doi: 10.1159/000495868.
3. Ou ZL, Luo Z, Wei W, Liang S, Gao TL, Lu YB. Hypoxia-induced shedding of MICA and HIF1A-mediated immune escape of pancreatic cancer cells from NK cells: role of circ_0000977/miR-153 axis. *RNA Biology*. (2019) 16: 1592-603. doi: 10.1080/15476286.2019.1649585.
4. Li J, Li ZH, Jiang P, Peng MJ, Zhang X, Chen K, et al. Circular RNA IARS (circ-IARS) secreted by pancreatic cancer cells and located within exosomes regulates endothelial monolayer permeability to promote tumor metastasis. *Journal of Experimental & Clinical Cancer Research*. (2018) 3: 177. doi: 10.1186/s13046-018-0822-3.
5. [Shi](https://pubmed.ncbi.nlm.nih.gov/?term=Shi+H&cauthor_id=32193152) HJ, [Li](https://pubmed.ncbi.nlm.nih.gov/?term=Li+H&cauthor_id=32193152) [H](https://pubmed.ncbi.nlm.nih.gov/32193152/#affiliation-1), [Zhen](https://pubmed.ncbi.nlm.nih.gov/?term=Zhen+T&cauthor_id=32193152) TT, Dong Y, Pei XJ, Zhang XL. hsa_circ_001653 implicates in the development of pancreatic ductal adenocarcinoma by regulating microRNA-377-mediated HOXC6 axis. *Mol Ther Nucleic Acids*. (2020) 20: 252-64. doi: 10.1016/j.omtn.2019.12.028.
6. Hao LG, Rong W, Bai LJ, Cui HS, Zhang SL, Li YC, et al. Upregulated circular RNA circ_0007534 indicates an unfavorable prognosis in pancreatic ductal adenocarcinoma and regulates cell proliferation, apoptosis, and invasion by sponging miR-625 and miR-892b. *J Cell Biochem*. (2019) 120: 3780-89. doi: 10.1002/jcb.27658.
7. Yao J, Zhang C, Chen YF, Gao SG. Downregulation of circular RNA circ-LDLRAD3 suppresses pancreatic cancer progression through miR-137-3p/ PTN axis. *Life Sci*. (2019) 239: 116871. doi: 10.1016/j.lfs.2019.116871.
8. [Huang](https://pubmed.ncbi.nlm.nih.gov/?term=Huang+L&cauthor_id=33425718) [LS](https://pubmed.ncbi.nlm.nih.gov/33425718/#affiliation-1), [Han](https://pubmed.ncbi.nlm.nih.gov/?term=Han+J&cauthor_id=33425718) [JX](https://pubmed.ncbi.nlm.nih.gov/33425718/#affiliation-2), [Yu](https://pubmed.ncbi.nlm.nih.gov/?term=Yu+H&cauthor_id=33425718) [HF](https://pubmed.ncbi.nlm.nih.gov/33425718/#affiliation-2), [Liu](https://pubmed.ncbi.nlm.nih.gov/?term=Liu+J&cauthor_id=33425718) [JL](https://pubmed.ncbi.nlm.nih.gov/33425718/#affiliation-2), [Gui](https://pubmed.ncbi.nlm.nih.gov/?term=Gui+L&cauthor_id=33425718) LL, [Wu](https://pubmed.ncbi.nlm.nih.gov/?term=Wu+Z&cauthor_id=33425718) [ZK](https://pubmed.ncbi.nlm.nih.gov/33425718/#affiliation-2), et al. CircRNA_000864 upregulates B-cell translocation gene 2 expression and represses migration and invasion in pancreatic cancer cells by binding to miR-361-3p. [*Front Oncol*](https://www.ncbi.nlm.nih.gov/pmc/articles/PMC7793745/)*.* (2020) 10: 547942. doi: [10.3389/fonc.2020.547942](https://dx.doi.org/10.3389%2Ffonc.2020.547942)
