# Supplementary material for: Research Progress of Circular RNA in Gastrointestinal Tumors
Source: Front Oncol. 2021 Apr 15;11:665246. doi: 10.3389/fonc.2021.665246 (PMC8082141; doi:10.3389/fonc.2021.665246)
Supplement: Supplementary file 5 [file Table_5.docx]

**Supplementary Table 5 Circular RNAs in hepatocellular carcinoma (HCC).**

| circRNAs | expression | mechanisms | target gene | function（promote +, suppress -) | Refs. |
| --- | --- | --- | --- | --- | --- |
| circcSMARCA5 | down | sponge miR-17-3p/  miR-181b-5p | TIMP3 | proliferation (-), migration (-). | [1] |
| hsa_circ_0016788 | up | sponge miR-486 | CDK4 | proliferation (+), migration (+), invasion (+), apoptosis (-). | [2] |
| circFBXO11 | up | sponge miR-605 | FOXO3/  ABCB1 | proliferation (+), cell cycle (+), OXA resistance (+). | [3] |
| circMET | up | sponge miR-30-5p | Snail / DPP4 / CXCL10 | EMT (+), immunosuppressive tumor microenvironment (+). | [4] |
| circUHRF1 | up | miR-449c-5p | TIM-3 | immune escape (+)，resistance of anti-PD1 immunotherapy (+). | [5] |
| circ-DB | up (in plasma exosomes of HCC patients with high body fat rate) | sponge miR-34a | USP/  Cyclin A2 | proliferation (+), cell DNA damage (-). | [6] |
| hsa_circ_100338 | up | sponge miR-141-3p | ZEB1 | proliferation (+). | [7] |
| [circNFATC3](http://www.chinapubmed.net/32667692) | down | sponge miR-548g | NFATC3 | proliferation (-), migration (-), invasion(-). | [8] |
| circMAST1 | up | sponge miR-1299 | CTNND1 | proliferation (+), migration (+), invasion (+). | [9] |
| circZNF609 | up | sponge miR-15a-5p/  miR-15b-5p | GLI2 | proliferation (+), migration (+), invasion (+), stemness (+), apoptosis(-). | [10] |
| circ_0001178 | up | sponge miR-382 | VEGFA | proliferation (+), migration (+), invasion (+). | [11] |
| hsa_circ_0091581 | up | sponge miR-526b | c-Myc | proliferation (+). | [12] |
| hsa_circ_0000092 | up | sponge miR-338-3p | HN1 | proliferation (+), migration (+), invasion (+), angiogenesis (+). | [13] |
| circMAN2B2 | up | sponge miRNA-217 | MAPK1 | proliferation (+). | [14] |
| circASAP1 | up | sponge miR-326/ miR-532-5p | MAPK1 / CSF-1 | proliferation (+), migration (+), invasion (+). | [15] |
| circRNA-5692 | down | sponge miR-328-5p | DAB2IP | proliferation (-), migration (-), invasion (-). | [16] |
| circRNAs | expression | mechanisms | target gene | function（promote +, suppress -) | Refs. |
| circ-FOXP1 | up | sponge miR-875-3p and miR-421 | SOX9 | proliferation (+), invasion (+), apoptosis (-). | [17] |
| circ_0015756 | up | sponge miR-7 | FAK | proliferation (+), migration (+), invasion (+), apoptosis (-). | [18] |
| hsa_circRNA_103809 | up | sponge miR-377-3p | FGFR1/  ERK | proliferation (+), cell cycle (+), migration (+). | [19] |
| circRNA-104718 | up | sponge miR-218-5p | TXNDC5 | proliferation (+), migration (+), invasion (+), apoptosis(-). | [20] |
| circHIAT1 | down | sponge miR-3171 | PTEN | proliferation (-). | [21] |
| hsa_circ_0101432 | up | sponge  miR-1258/ miR-622 | MAPK1 | proliferation (+), migration (+), invasion (+), apoptosis(-). | [22] |
| circ_0005075 | up | sponge miR-335 | MAPK2 | proliferation (+), migration (+), invasion (+), apoptosis(-), chemotherapy resistance (+). | [23] |
| circ_0000267 | up | sponge miR-646 | - | proliferation (+), migration (+), invasion (+), apoptosis(-). | [24] |
| circSETD3 | down | sponge miR-421 | MAPK14 | proliferation (-), G1/S arrest (+). | [25] |
| circADAMTS14 | down | sponge miR-572 | RCAN1 | proliferation (-), invasion (-), apoptosis(+). | [26] |
| circBIRC6 | up | sponge miR-3918 | Bcl2 | proliferation (+), migration (+), invasion (+), apoptosis (-). | [27] |
| hsa_circ_0103809 | up | sponge miR-490-5p | SOX2 | proliferation (+), migration (+), apoptosis (-). | [28] |
| circRNA-101368 | up | sponge miR-200a | HMGB1/  RAGE | migration(+), invasion(+). | [29] |
| circMTO1 | down | sponge miR-9 | p21 | proliferation (-), invasion (-). | [30] |
| circMYLK | up | sponge miR-362-3p | Rab23 | proliferation (+), migration (+), invasion (+). | [31] |
| circRNAs | expression | mechanisms | target gene | function（promote +, suppress -) | Refs. |
| circZFR | up | sponge miR-3619-5p | CTNNB1/  Wnt/β-catenin | proliferation (+), EMT (+). | [32] |
| hsa_circ_101280 | up | sponge miR-375 | JAK2 | proliferation (+), apoptosis (-). | [33] |
| circADAMTS13 | down | sponge miR-484 | - | proliferation (-). | [34] |
| hsa_circ_0005785 | up | sponge miR-578 | APRIL | proliferation (+), migration (+), invasion (+)，apoptosis (-). | [35] |
| circLARP4 | down | sponge miR-761 | RUNX3/p53/  p21 | proliferation (-), mediated cell cycle arrest and induced senescence. | [36] |
| circGSK3B | up | sponge miR-1265 | CAB39 | proliferation (+), migration (+), invasion (+). | [37] |
| circ-MALAT1 | up | sponge miR-6887-3p  and form an ternary complex with both ribosomes and PAX5 mRNA | PAX5,  JAK2 | self-renewal of hepatocellular cancer stem cells (+). | [38] |
| circRHOT1 | up | interact with TIP60 | NR2F6 | proliferation (+), migration (+), invasion (+). | [39] |
| circBACH1 | up | interact with  HuR | P27 | cell activity (+), proliferation (+), G1/S phase cell cycle (+). | [40] |
| circRNA-100338 | up | transfer exosomal circRNA-100338 to recipient HUVECs | HUVECs | proliferation (+), angiogenesis (+), permeability (+), vasculogenic mimicry (VM) formation ability of HUVEC (+),  tumor metastasis (+). | [41] |
| circZKSCAN1 | down | sponge FMRP | CCAR1 | proliferation (-). | [42] |
| circβ-catenin | up | encode β-catenin-370aa | GSK3β/ β-catenin | proliferation (+), migration (+), invasion (+). | [43] |

# Supplementary Table 5 Reference

1. Yu J, Xu QG, Wang ZG, Yang Y, Zhang L, Ma JZ, et al. Circular RNA cSMARCA5 inhibits growth and metastasis in hepatocellular carcinoma. *Journal of Hepatology*. (2018) 68: 1214-27. doi: 10.1016/j.jhep.2018.01.012.
2. Guan Z, Tan J, Gao W, Li X, Yang YD, Li XG, et al. Circular RNA hsa_circ_0016788 regulates hepatocellular carcinoma tumorigenesis through miR-486/CDK4 pathway. *Journal of Cellular Physiology*. (2018) 234: 500-8. doi: 10.1002/jcp.26612.
3. Li J, Qin XP, Wu RS, Wan L, Zhang L, Liu R. Circular RNA circFBXO11 modulates hepatocellular carcinoma progress and oxaliplatin resistance through miR-605/FOXO3/ABCB1 axis. *J Cell Mol Med*. (2020) 9: 5152-61. doi: 10.1111/jcmm.15162.
4. Huang XY, Zhang PF, Wei CY, Peng R, Lu JC, Gao C, et al. Circular RNA circMET drives immunosuppression and anti-PD1 therapy resistance in hepatocellular carcinoma via the miR-30-5p/snail/DPP4 axis. *Mol Cancer*. (2020) 19: 92. doi: 10.1186/s12943-020-01213-6.
5. Zhang PF, Gao C, Huang XY, Lu JC, Guo XJ, Shi GM, et al. Cancer cell-derived exosomal circUHRF1 induces natural killer cell exhaustion and may cause resistance to anti-PD1 therapy in hepatocellular carcinoma. *Mol Cancer*. (2020) 19: 110. doi: 10.1186/s12943-020-01222-5.
6. Zhang HY, Deng T, Ge SH, Liu Y, Bai M, Zhu KG, et al. Exosome circRNA secreted from adipocytes promotes the growth of hepatocellular carcinoma by targeting deubiquitination-related USP7. *Oncogene*. (2019) 38: 2844-59. doi: 10.1038/s41388-018-0619-z.
7. [Cheng XL,](http://www.chinapubmed.net/search/?author=Xiaoli%20Cheng)[Tian P,](http://www.chinapubmed.net/search/?author=Pan%20Tian)[Zheng](http://www.chinapubmed.net/search/?author=Wengzhong%20Zheng) WZ, Yan XT. Piplartine attenuates the proliferation of hepatocellular carcinoma cells via regulating hsa_circ_100338 expression. *Cancer Medcine*. (2020) 9: 4265-73. doi: 10.1002/cam4.3043.
8. Jia CC, Yao ZC, Lin ZX, Zhao LY, Cai XR, Chen SH, et al. circNFATC3 sponges miR‐548I acts as a ceRNA to protect NFATC3 itself and suppressed hepatocellular carcinoma progression. *J Cell Physiol*. (2020) 236: 1252-69. doi:10.1002/jcp.29931.
9. Yu XF, Sheng P, Sun J, Zhao XJ, Zhang JT, Li YY, et al. The circular RNA circMAST1 promotes hepatocellular carcinoma cell proliferation and migration by sponging miR-1299 and regulating CTNND1 expression. *Cell Death Dis*. (2020) 11: 340. doi: 10.1038/s41419-020-2532-y.
10. He YK, Huang H, Jin L, Zhang F, Zeng M, Wei L, et al. CircZNF609 enhances hepatocellular carcinoma cell proliferation, metastasis, and stemness by activating the Hedgehog pathway through the regulation of miR-15a-5p/15b-5p and GLI2 expressions. *Cell Death Dis*. (2020) 11: 358. doi:10.1038/s41419-020- 2441-0.
11. Gao S, Hu W, Huang X, Huang XY, Chen WW, Hao LD, et al. Circ_0001178 regulates miR-382/VEGFA axis to facilitate hepatocellular carcinoma progression. *Cell Signal*. (2020) 72: 109621. doi: 10.1016/j.cellsig.2020.109621.
12. Wei XL, Zheng WJ, Tian PK, He Y, Liu H, Peng MJ, et al. Oncogenic hsa_circ_0091581 promotes the malignancy of HCC cell through blocking miR-526b from degrading c-MYC mRNA. *Cell Cycle*. (2020) 19: 817-24. doi: 10.1080/15384101.2020.1731945.
13. [Pu](https://pubmed.ncbi.nlm.nih.gov/?term=Pu+J&cauthor_id=32077624) J, [Wang](https://pubmed.ncbi.nlm.nih.gov/?term=Wang+J&cauthor_id=32077624) JC, [Li](https://pubmed.ncbi.nlm.nih.gov/?term=Li+W&cauthor_id=32077624) WC, Lu Y, Wu XJ, Long XD, et al. hsa_circ_0000092 promotes hepatocellular carcinoma progression through up-regulating HN1 expression by binding to microRNA-338-3p. *J Cell Mol Med*. (2020) 2: 15010. doi: 10.1111/jcmm.15010.
14. Fu XY, Zhang JJ, He X, Yan X, Wei J, Huang M, et al. Circular RNA MAN2B2 promotes cell proliferation of hepatocellular carcinoma cells via the miRNA-217/ MAPK1 axis. *J Cancer*. (2020) 11:3318-26. doi: 10.7150/jca.36500.
15. Hu ZQ, Zhou SL, Li J, Zhou ZJ, Wang PC, Xin HY, et al. Circular RNA sequencing identifies circASAP1 as a key regulator in hepatocellular carcinoma metastasis. *Hepatology*. (2019) 72: 906-22. doi: 10.1002/hep.31068.
16. Liu ZG, Yu YQ, Huang ZB, Kong Y, Hu XW, Xiao W, et al. CircRNA-5692 inhibits the progression of hepatocellular carcinoma by sponging miR-328-5p to enhance DAB2IP expression. *Cell Death Dis*. (2019) 10: 900. doi:10.1038/s41419-019-2089-9.
17. Wang W, Li Y, Li XL, Liu BW, Han SY, Li XF, et al. Circular RNA circ-FOXP1 induced by SOX9 promotes hepatocellular carcinoma progression via sponging miR-875-3p and miR-421. *Biomedicine & Pharmacotherapy*. (2020) 121: 109517. doi: 10.1016/j.biopha.2019.109517.
18. Liu L, Yang X, Li NF, Lin L, Luo H. Circ_0015756 promotes proliferation, invasion and migration by microRNA-7-dependent inhibition of FAK in hepatocellular carcinoma. *Cell Cycle.* (2019) 18: 2939-53. doi:10.1080/15384101. 2019.1664223.
19. Zhan W, Liao X, Chen ZS, Li LH, Tian T, Yu L, et al. Circular RNA hsa_circRNA_103809 promoted hepatocellular carcinoma development by regulating miR‐377‐ 3p/FGFR1/ERK axis. *Journal of Cellular Physiology*. (2019): 1-13. doi: 10.1002/jcp.29092.
20. Yu JZ, Yang MJ, Zhou B, Luo JJ, Zhang ZH, Zhang W, et al. CircRNA-104718 acts as competing endogenous RNA and promotes hepatocellular carcinoma progression through microRNA-218-5p/TXNDC5 signaling pathway. *Clinical Science*. (2019) 133: 1487-503. doi: 10.1042/CS20190394.
21. Wang ZM, Zhao YX, Wang Y, Jin CX. Circular RNA circHIAT1 inhibits cell growth in hepatocellular carcinoma by regulating miR-3171/PTEN axis. *Biomedicine & Pharmacotherapy.* (2019) 116:108932. doi:10.1016/j. biopha.2019. 108932.
22. Zou HB, Xu XG, Luo LY, Zhang Y, Luo L, Yao YT, et al. Hsa_circ_0101432 promotes the development of hepatocellular carcinoma (HCC) by adsorbing miR-1258 and miR-622. *Cell Cycle.* (2019) 18: 2398-413. doi: 10.1080/15384101. 2019.1618120.
23. Yang XY, Song H, Zi ZG, Kou JT, Chen SH, Dai Y, et al. Circ_0005075 promotes hepatocellular carcinoma progression by suppression of microRNA-335. *Journal of Cellular Physiology*. (2019) 234: 21937-946. doi: 10.1002/jcp.28757.
24. Pan HL, Tang L, Jiang H, Li XY, Wang RL, Gao JX, et al. Enhanced expression of circ_0000267 in hepatocellular carcinoma indicates poor prognosis and facilitates cell progression by sponging miR‐646. *Journal of Cellular Biochemistry*. (2019): 1-8. doi: 10.1002/jcb.28411.
25. Xu LL, Feng XF, Hao XY, Wang P, Zhang YF, Zheng XB, et al. CircSETD3 (Hsa_circ_0000567) acts as a sponge for microRNA-421 inhibiting hepatocellular carcinoma growth. *J Exp Clin Cancer Res*. (2019) 38: 98. doi: 10.1186/s13046-019-1041-2.
26. Song CL, Li DQ, Liu HY, Sun HY, Liu Z, Zhang LR, et al. The competing endogenous circular RNA ADAMTS14 suppressed hepatocellular carcinoma progression through regulating microRNA-572/regulator of calcineurin 1. *J Cell Physiol*. (2019) 234: 2460-70. doi: 10.1002/jcp.26764.
27. Yang GS, Wang X, Liu BQ, Lu ZH, Xu ZZ, Xiu P, et al. circ-BIRC6, a circular RNA, promotes hepatocellular carcinoma progression by targeting the miR-3918/ Bcl2 axis. *Cell Cycle*. (2019) 18: 976-89. doi:10.1080/15384101.2019.1601477.
28. Cai HJ, Hu BR, Ji L, Ruan XJ, Zheng ZH. Hsa_circ_0103809 promotes cell proliferation and inhibits apoptosis in hepatocellular carcinoma by targeting miR-490-5p/SOX2 signaling pathway. *Am J Transl Res*. (2018) 10: 1690-702.
29. Li SL, Gu HM, Huang Y, Peng Q, Zhou RR, Yi PP, et al. Circular RNA 101368/miR-200a axis modulates the migration of hepatocellular carcinoma through HMGB1/RAGE signaling. *Cell Cycle*. (2018) 17: 2349-59. doi: 10.1080/15384101.2018.1526599.
30. Han D, Li JX, Wang HM, Su XP, Hou J, Gu Y, et al. Circular RNA circMTO1 acts as the sponge of microRNA-9 to suppress hepatocellular carcinoma progression. *Hepatology*. (2017) 66: 1151-64. doi: 10.1002/hep.29270.
31. Li ZQ, Hu YS, Zeng QL, Wang HY, Yan JY, Li H, et al. Circular RNA MYLK promotes hepatocellular carcinoma progression by increasing Rab23 expression by sponging miR-362-3p. *Cancer Cell International*. (2019) 19: 211. doi: 10.1186/s12935-019-0926-7.
32. Tan AC, Li QX, Chen LZ. CircZFR promotes hepatocellular carcinoma progression through regulating miR-3619–5p/CTNNB1 axis and activating Wnt/β-catenin pathway. *Archives of Biochemistry and Biophysics*. (2018) 661: 196-202. doi: 10.1016/j.abb.2018.11.020.
33. Cao S, Wang GH, Wang J, Li C, Zhang L. Hsa_circ_101280 promotes hepatocellular carcinoma by regulating miR-375/JAK2. *Immunology and Cell Biology*. (2019) 97: 218. doi: 10.1111/imcb.12213.
34. Qiu LM, Huang YB, Li ZL, Dong XQ, Chen G, Xu HP, et al. Circular RNA profiling identifies circ ADAMTS13 as a miR484 sponge which suppresses cell proliferation in hepatocellular carcinoma. *Molecular Oncology*. (2019) 13: 441-55. doi: 10.1002/1878-0261.12424.
35. Wu AQ, Li Y, Kong MZ, Zhu BH, Liu RY, Bao F, et al. Upregulated hsa_circ_0005785 facilitates cell growth and metastasis of hepatocellular carcinoma through the miR-578/APRIL axis. *Front Oncol.* (2020) 10: 1388. doi:10.3389/fonc.2020.01388.
36. Chen ZQ, Zuo XL, Pu LY, [Zhang Y,](http://www.chinapubmed.net/search/?author=Yao%20Zhang)[Han GY,](http://www.chinapubmed.net/search/?author=Guoyong%20Han) [Zhang L,](http://www.chinapubmed.net/search/?author=Long%20Zhang)et al. circLARP4 induces cellular senescence through regulating miR-761/RUNX3/p53/p21 signaling in hepatocellular carcinoma. *Cancer Sci*. (2019) 110: 568-81. doi: 10.1111/cas.13901.
37. Li K, Cao JC, Zhang ZT, Chen KY, Ma TL, Yang WJ, et al. Circular RNA circGSK3B promotes cell proliferation, migration, and invasion by sponging miR-1265 and regulating CAB39 expression in hepatocellular carcinoma. *Front Oncol*. (2020) 10: 598256. doi:10.3389/fonc.2020.598256.
38. Chen L, Kong RJ, Wu C, Wang S, Liu ZX, Liu SP, et al. Circ‐MALAT1 functions as both an mRNA translation brake and a microRNA sponge to promote self‐renewal of hepatocellular cancer stem cells. *Advanced Science*. (2020) 7: 1900949. doi: 10.1002/advs.201900949.
39. Wang LY, Long HY, Zheng QH, Bo XT, Xiao XH, Li B. Circular RNA circRHOT1 promotes hepatocellular carcinoma progression by initiation of NR2F6 expression. *Mol Cancer*. (2019) 18: 119. doi: 10.1186/s12943-019-1046-7.
40. Liu BQ, Yang GS, Wang X, Liu JF, Lu ZH, Wang Q, et al. CircBACH1 (hsa_circ_0061395) promotes hepatocellular carcinoma growth by regulating p27 repression via HuR. [*J Cell Physiol*](http://www.chinapubmed.net/search/?f_journal=J%20Cell%20Physiol). (2020) 235: 6929-41. doi: 10.1002/jcp.29589.
41. Huang XY, Huang ZL, Huang J, Xu B, Huang XY, Xu YH, et al. Exosomal circRNA-100338 promotes hepatocellular carcinoma metastasis via enhancing invasiveness and angiogenesis. *J Exp Clin Cancer Res*. (2020) 39: 20. doi: 10.1186/s13046-020-1529-9.
42. Zhu YJ, Zheng B, Luo GJ, [Ma XH](http://www.chinapubmed.net/search/?author=Xu-Kai%20Ma), [Lu XY,](http://www.chinapubmed.net/search/?author=Xin-Yuan%20Lu) [Lin XM,](http://www.chinapubmed.net/search/?author=Xi-Meng%20Lin)et al. Circular RNAs negatively regulate cancer stem cells by physically binding FMRP against CCAR1 complex in hepatocellular carcinoma. *Theranostics*. (2019) 9: 3526-40. doi: 10.7150/thno.32796.
43. Liang WC, Wong CW, Liang PP, Shi M, Cao Y, Rao ST, et al. Translation of the circular RNA circβ-catenin promotes liver cancer cell growth through activation of the Wnt pathway. *Genome Biology*. (2019) 20: 84. doi: 10.1186/s13059-019-1685-4.
