# Supplementary material for: Research Progress of Circular RNA in Gastrointestinal Tumors
Source: Front Oncol. 2021 Apr 15;11:665246. doi: 10.3389/fonc.2021.665246 (PMC8082141; doi:10.3389/fonc.2021.665246)
Supplement: Supplementary file 6 [file Table_6.docx]

**Supplementary Table 6 Circular RNAs in gallbladder cancer.**

| circRNAs | expression | mechanisms | target gene | function（promote +, suppress -) | Refs. |
| --- | --- | --- | --- | --- | --- |
| circFOXP1 | up | interacted with PTBP1 and sponge miR-370 | PKLR | proliferation (+), migration (+), invasion (+), Warburg effect (+), apoptosis (-). | [1] |
| circERBB2 | up | regulate ribosomal DNA transcription | PA2G4/  TIFIA | proliferation (+). | [2] |

**Supplementary Table 6 Reference**

1. [Wang](https://pubmed.ncbi.nlm.nih.gov/?term=Wang+S&cauthor_id=31623628) SH, [Zhang](https://pubmed.ncbi.nlm.nih.gov/?term=Zhang+Y&cauthor_id=31623628) YJ, [Cai](https://pubmed.ncbi.nlm.nih.gov/?term=Cai+Q&cauthor_id=31623628) Q, [Ma](https://pubmed.ncbi.nlm.nih.gov/?term=Ma+M&cauthor_id=31623628) MZ, [Jin](https://pubmed.ncbi.nlm.nih.gov/?term=Jin+LY&cauthor_id=31623628) LY, [Weng](https://pubmed.ncbi.nlm.nih.gov/?term=Weng+M&cauthor_id=31623628) MZ, et al. Circular RNA FOXP1 promotes tumor progression and Warburg effect in gallbladder cancer by regulating PKLR expression. *Mol Cancer*. (2019) 18: 145. doi: 10.1186/s12943-019-1078-z.
2. Huang XC, He M, Huang S, Lin RR, Zhan M, Yang D, et al. Circular RNA circERBB2 promotes gallbladder cancer progression by regulating PA2G4-dependent rDNA transcription. *Mol Cancer*. (2019) 18: 166. doi: 10.1186/s12943-019-1098-8.
