# Supplementary material for: Research Progress of Circular RNA in Gastrointestinal Tumors
Source: Front Oncol. 2021 Apr 15;11:665246. doi: 10.3389/fonc.2021.665246 (PMC8082141; doi:10.3389/fonc.2021.665246)
Supplement: Supplementary file 7 [file Table_7.docx]

**Supplementary Table 7 Circular RNAs in cholangiocarcinoma.**

| circRNAs | expression | mechanisms | target gene | function（promote +, suppress -) | Refs. |
| --- | --- | --- | --- | --- | --- |
| circ-CCAC1 | up | sponge miR-514a-5p | YY1/  CAMLG | proliferation (+), migration (+), invasion (+). | [1] |

**Supplementary Table 7 Reference**

1. Xu Y, Leng KM, Yao Y, Kang PC, Liao GQ, Han Y, et al. A novel circular RNA, circ-CCAC1, contributes to CCA progression, induces angiogenesis, and disrupts vascular endothelial barriers. *Hepatology*. (2020) 8: 31493. doi:10.1002/hep.

31493.
